# HIV Provirus Stably Reproduces Parental Latent and Induced Transcription Phenotypes Regardless of the Chromosomal Integration Site

**DOI:** 10.1128/JVI.02842-15

**Published:** 2016-05-12

**Authors:** Farhad B. Hashemi, Kris Barreto, Wendy Bernhard, Pargol Hashemi, Adam Lomness, Ivan Sadowski

**Affiliations:** Biochemistry and Molecular Biology, Molecular Epigenetics, Life Sciences Institute, University of British Columbia, Vancouver, BC, Canada

## Abstract

Understanding the mechanisms of HIV proviral latency is essential for development of a means to eradicate infection and achieve a cure. We have previously described an *in vitro* latency model that reliably identifies HIV expression phenotypes of infected cells using a dual-fluorescence reporter virus. Our results have demonstrated that ∼50% of infected cells establish latency immediately upon integration of provirus, a phenomenon termed early latency, which appears to occur by mechanisms that are distinct from epigenetic silencing observed with HIV provirus that establishes productive infections. In this study, we have used a mini-dual HIV reporter virus (mdHIV) to compare the long-term stability of provirus produced as early latent or productive infections using Jurkat-Tat T cell clones. Cloned lines bearing mdHIV provirus integrated at different chromosomal locations display unique differences in responsiveness to signaling agonists and chromatin-modifying compounds, and they also produce characteristic expression patterns from the 5′ long terminal repeat (LTR) dsRed and internal EIF1α-enhanced green fluorescent protein (EIF1α-eGFP) reporters. Furthermore, reporter expression profiles of single cell sorted subcultures faithfully reproduce expression profiles identical to that of their original parental population, following prolonged growth in culture, without shifting toward expression patterns resembling that of cell subclones at the time of sorting. Comparison of population dispersion coefficient (CV) and mean fluorescence intensity (MFI) of the subcloned lines showed that both untreated and phorbol myristate acetate (PMA)-ionomycin-stimulated cultures produce expression patterns identical to those of their parental lines. These results indicate that HIV provirus expression characteristics are strongly influenced by the epigenetic landscape at the site of chromosomal integration.

**IMPORTANCE** There is currently considerable interest in development of therapies to eliminate latently infected cells from HIV-infected patients on antiretroviral therapy. One proposed strategy, known as “shock and kill,” would involve treatment with therapies capable of inducing expression of latent provirus, with the expectation that the latently infected cells could be killed by a host immune response or virus-induced apoptosis. In clinical trials, histone deacetylase (HDAC) inhibitors were shown to cause reactivation of latent provirus but did not produce a significant effect toward eliminating the latently infected population. Results shown here indicate that integration of HIV provirus at different chromosomal locations produces significant effects on the responsiveness of virus expression to T cell signaling agonists and chromatin-modifying compounds. Given the variety of phenotypes produced by integrated provirus, it is unlikely that any single potential shock-and-kill therapy will be effective toward purging the latently infected population.

## INTRODUCTION

Human immunodeficiency virus (HIV) infection is presently incurable because the virus establishes latent infections of resting memory immune cells in which provirus expression has become transcriptionally silenced (1). Despite the successes of antiretroviral therapy (ART) in suppressing HIV replication, the latently infected cells persist in asymptomatic patients and act as a reservoir for rebound of HIV viremia when ART is discontinued (2). Due to the latent HIV reservoir's stability and long half-life, it presents a major barrier to eradication of infection (3). Thus, there is currently significant interest in development of strategies, such as “shock and kill,” to purge the latent HIV reservoir in infected patients (1).

Multiple layers of regulation contribute to establishment of latent HIV provirus. HIV-1 predominately integrates into actively transcribed chromosomal regions (4–6), and transcriptionally active provirus gradually shuts down in unstimulated T cells, regardless of integration site, indicating that establishing latency involves mechanisms intrinsic to the HIV 5′ long terminal repeat (LTR) itself (7). Transcriptional activators bound to the HIV LTR, regulated by T cell receptor signaling, are turned off in cells that revert to a resting state and in many instances are replaced by repressors that recruit histone deacetylases (HDACs) (8). Histone deacetylation is accompanied by positioning of two nucleosomes on the LTR near the transcriptional start site and immediately upstream of the enhancer region, known as nuc-1 and nuc-0, respectively (9). Multiple factors that bind to the transcriptionally repressed HIV LTR can recruit the Suv39H1, EZH2, and G9a histone methyl transferases that catalyze trimethylation of histone H3, which, in turn, causes recruitment of heterochromatin protein 1 (HP1) and PRC2 to promote spreading of repressive epigenetic marks to adjacent nucleosomes (10–13). Resting CD4^+^ T cells also express several microRNAs (miRNAs), which target the 3′ end of HIV-1 transcripts that can contribute to transcriptional silencing of the provirus (14). This combination of epigenetic silencing mechanisms eventually causes shutdown of viral transcription (15). An important consequence of repressed HIV-1 expression is the loss of the viral transcriptional activator Tat, which leads to disruption of its strong positive-feedback loop (16). The cellular target for Tat, the elongation factor pTEFb, is also downregulated in resting CD4^+^ T cells by decreased cyclin T1 expression and reduced phosphorylation of Cdk9 at the activation loop (17). A combination of these mechanisms in T cells causes gradual shutdown of provirus to produce latently infected cells (18).

Using a dual-reporter HIV derivative that allows detection of infected cells independent of LTR activity, we have shown that a significant fraction of cells harbor latent HIV provirus immediately upon infection (19). While these findings were confirmed by others (20), they also support earlier reports showing a low frequency of latent provirus in the absence of prior viral gene expression (21). The proportion of cells that establish early latency can be altered by manipulation of NF-κB activity (22) and is associated with the binding of YY1 to the 5′ LTR (23). Establishment of early latency is not influenced by the site of integration, as determined by comparing integration sites in populations of cells that have established latent or productive infections at 24 h postinfection (22). We have proposed that the basal cellular activation state may influence signaling pathways controlling HIV LTR activation and dictate a decision to establish either latent or productive HIV infections (22).

Despite the fact that the site of integration does not seem to influence establishment of latency, several observations indicate that the chromosomal integration site can dictate HIV expression phenotype. Specifically, integration into genes associated with cell growth control seems to confer an advantage for persistence of latent provirus in patients treated with antiretroviral therapy (24). Another study reported that latently infected T cells undergoing clonal expansion predominately harbor either defective provirus or replication-competent virus integrated near transcriptionally silenced regions of the genome (25, 26). The site of integration also affects viral burst size in clonal populations of cells bearing mini-HIV green fluorescent protein (GFP) reporter virus (27). Integration site may also influence responsiveness of HIV provirus to a variety of T cell signaling agonists and chromatin-modifying agents (26, 28). However, most of these studies have not determined whether chromosomal location affects expression phenotypes of latent provirus that is established early upon infection, because HIV reporter viruses used in these studies detected infection by LTR activity.

In this study, we have examined the stability of HIV basal and induced expression phenotypes in T cell lines with latent provirus, where infection was identified independent of HIV LTR activity, over prolonged periods of culture. We found that clones representing a broad range of HIV basal and induced expression phenotypes are highly stable, and faithfully recapitulate the parental phenotypes following months of culture. Furthermore, we found that various clones bearing integrated provirus produce dramatically different responses to signaling agonists and chromatin-modifying compounds. These observations have significant implications for potential shock-and-kill therapies, because of the differences in expression phenotypes associated with various sites of provirus integration.

## MATERIALS AND METHODS

### Cell and virus cultures.

**FIG 1:**
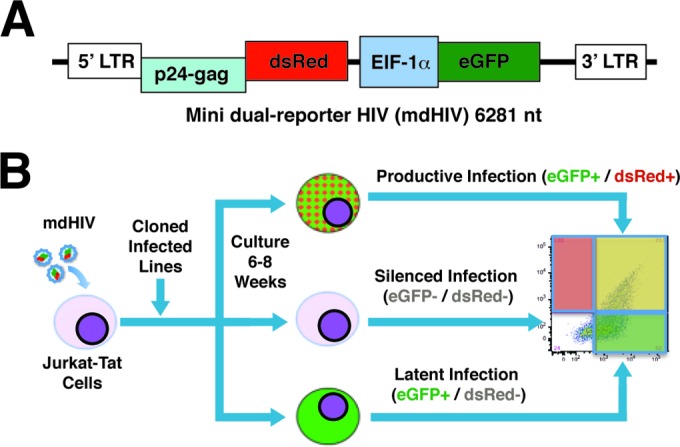
(A) Schematic representation of the mini-dual-HIV (mdHIV) reporter virus. Infected cells can be detected by expression of eGFP from an internal EIF1α promoter, while dsRed is expressed from the HIV 5′ LTR. (B) Jurkat-Tat cells were infected with mdHIV, and infected (eGFP^+^) single cell clones were isolated and expanded. Following expansion in culture for 8 weeks, provirus infected cell lines generate populations of cells expressing both eGFP and dsRed (productive infection), populations expressing EIF1α-eGFP (latent infection), or cells in which both the EIF1α and HIV LTR promoters have been silenced (silenced infection), which can be detected by flow cytometry.

Jurkat-J6 and Jurkat-Tat cells were obtained through the NIH AIDS Research and Reference Reagent Program, Division of AIDS, NIAID, NIH: Jurkat-Tat cells from Antonella Caputo, William Haseltine, and Joseph Sodroski. Human embryonic kidney 293T (HEK293T) cells were obtained from the American Type Culture Collection. Jurkat cells were grown in RPMI 1640 (Sigma) supplemented with 10% fetal bovine serum (Sigma), penicillin (100 U/ml), streptomycin (100 μg/ml), and amphotericin B (Fungizone; 0.25 μg/ml) (Gibco). Jurkat-Tat cells were grown in the same with an additional supplement of Geneticin (0.8 mg/ml; Invitrogen). Cell cultures were maintained in a humidified 37°C incubator with a 5% CO_2_ atmosphere. Cells were stimulated with 50 nM phorbol 12-myristate 13-acetate (PMA; Sigma) alone or in combination with ionomycin (IO; 1 μM; Sigma) for 24 h where indicated below. HEK293T cells were cultured in Dulbecco modified Eagle medium (DMEM; Sigma) with 10% fetal bovine serum supplemented with penicillin (100 U/ml), streptomycin (100 U/ml), and amphotericin B (0.25 μg/ml; Life Technologies). The mini-dual-fluorescence HIV (mdHIV) reporter virus expresses dsRed from the 5′ HIV LTR and enhanced GFP (eGFP) from an internal EIF1α promoter to enable detection of infected cells independently from LTR expression (Fig. 1A) (23). The RGH2 (PGK-mCherry) reporter virus (see Fig. S7 in the supplemental material) was as described previously (19). Vesicular stomatitis virus G protein (VSV-G)-pseudotyped mdHIV or RGH2 reporter virus was generated by cotransfection of HEK293T cells, as described previously (19, 23). Viral stocks were purified through a 0.2-μm Whatman Puradisc syringe filter and concentrated by centrifugation in Amicon Ultra-4 centrifugal filter units (Millipore).

### Fluorescence-activated cell sorting (FACS).

Approximately 1 × 10^6^ Jurkat-Tat cells were infected at a multiplicity of infection (MOI) of ∼0.1 for 1 h. Infected Jurkat-Tat cells were monitored for eGFP and dsRed expression every day for 4 days and once weekly thereafter for a month, using a BD Biosciences LSR II system as previously described (23). The LSR II was equipped with 488-nm and 561-nm lasers, which enable separate detection of eGFP and dsRed expression with minimal compensation (29). Threshold of forward scatter (FSC) and side scatter (SSC) were set so that only live cells were counted and sorted. Clones were isolated by live sorting into 96-well plates containing 100 μl of cell-free RPMI 1640 that had been incubated with untreated Jurkat-Tat cells for 2 days. Following expansion, the clones were analyzed by two-color sorting to measure expression of HIV-LTR (dsRed) and the internal EIF1α promoter (eGFP), as a marker for proviral integration. The mean fluorescence intensity (MFI) and CV (coefficient of variation) were obtained using FlowJo analysis software (v9.5.2; Tree Star). Robust CV indicating dispersion coefficient of cellular populations was determined by FlowJo using the following formula: 100 × [1/2] (intensity at 84.13% − intensity at 15.87%)/median.

### Identification of HIV integration sites.

Integration sites of mdHIV latently infected Jurkat-Tat clones were determined by a restriction enzyme PCR strategy using the scheme shown in Fig. S4 in the supplemental material and as previously described (22). Briefly, 2 μg of genomic DNA from 2 × 10^7^ cells was digested with MseI and ligated to 0.5 μg of annealed adaptor oligonucleotides. The products were subsequently digested with BglII, and the HIV LTR-flanking DNA fragments were amplified by nested PCR with adaptor-specific primers WB214 and WB216, followed by adaptor- and LTR-specific primers WB213 and WB215 (Table 1). Amplicons were purified on agarose gels and sequenced (Eurofin MWG OperonR, Louisville, KY) using the HIV-specific primer WB213. PCR fragments that did not yield sequence reads were reamplified with oligonucleotides IS2091 and IS2092 (Table 1), digested with BamHI and HindIII, and cloned into pGEM3Zf(−). Clones from the ligation were sequenced with the pGEM3Zf(−)-specific primers IS2137 and IS2138. LTR-flanking DNA sequences were analyzed using the USC human genome browser (http://genome.ucsc.edu).

**TABLE 1:** Oligonucleotides used in this study

Name	Use	Sequence
WB213	LTR-specific 1	CTTAAGCCTCAATAAAGCTTGCCTTGAG
WB214	MseI linker 1	GTAATACGACTCACTATAGGGC
WB215	LTR-specific 2	AGACCCTTTTAGTCAGTGTGGAAAATC
WB216	MseI linker 2	AGGGCTCCGCTTAAGGGAC
IS2091	Linker F-BamHI	CCGGGATCCAGACCCTTTTAGTCAGTGTGGAAAATC
IS2092	Linker R-HindIII	GGCAAGCTTAGGGCTCCGCTTAAGGGAC
IS2137	pGEM3Z F	TCTTCGCTATTACGCCAGCTGGCGAA
IS2138	pGEM3Z R	GCTTTACACTTTATGCTTCCGGCTC

### Transient-transfection and luciferase reporter assays.

The HIV-1 LTR-luciferase reporter gene (TAR^+^) plasmid construct was described previously (30). Jurkat-Tat clonal cell lines bearing mdHIV reporter virus were transfected using Lipofectamine R2000 (Invitrogen) according to the manufacturer's instructions (31). Cells were transfected in 24-well plates, containing 1 × 10^6^ cells per well, with 1 μg of HIV-1 LTR-luciferase plasmid and 0.1 μg of respiratory syncytial virus (RSV) β-galactosidase (β-Gal) internal control plasmid. The cells were incubated for 48 h and exposed to PMA for 24 h prior to lysis of the cells and assay for luciferase and β-galactosidase activity (31). Results are presented as the means ± standard deviations (SD) from three independent transfected samples.

## RESULTS

### Clonal cell lines with mini-HIV reporter provirus produce distinct responses to signaling and chromatin-modifying agents.

**FIG 2:**
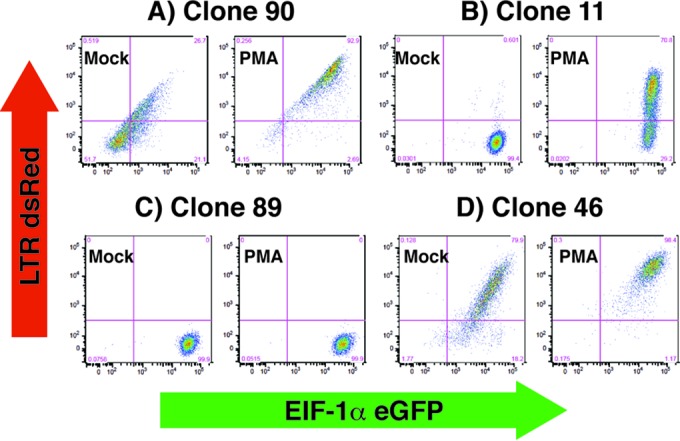
Clones (see Fig. S2 in the supplemental material) of Jurkat-Tat cells infected with mdHIV (eGFP^+^) were isolated by live sorting 48 h postinfection and expanded in culture for 4 weeks. Untreated samples (mock), or cells treated for 24 h with PMA, of each cloned line were analyzed by FACS for expression of dsRed from the 5′ LTR (vertical) and eGFP (horizontal) from the internal EIF1α promoter; results from four classes of representative clones are shown. Approximately 70% of the clones display silenced expression of both eGFP and dsRed in untreated cells, but both were inducible by PMA treatment (e.g., clone 90 in panel A). A total of 15% of the expanded clones were found to express eGFP but not dsRed in untreated cells (e.g., clones 11 and 89 in panels B and C), but approximately one-half of these induce dsRed in response to PMA (clone 11 in panel B). The remaining 15% of clones show constitutive expression of both eGFP and dsRed (e.g., clone 46 in panel D).

To characterize the effect that the site of chromosomal integration may have on response of HIV provirus to T cell signaling agonists and chromatin-modifying drugs, we isolated a panel of cell lines harboring integrants of a mini-dual-fluorescence HIV reporter virus (mdHIV), in which dsRed is expressed from the 5′ LTR and eGFP from an internal EIF1α promoter to enable detection of infected cells independently from LTR expression (Fig. 1A) (23). We used a minivirus to circumvent challenges of stably maintaining clonal cell lines infected with full-length replication-competent virus (19, 22), likely due to cytotoxic effects of HIV accessory proteins. However, because mdHIV does not express the viral transactivator Tat, it was necessary to provide Tat in *trans* for this objective, using the Jurkat-Tat cell line, in which Tat is expressed constitutively (32). With this virus, productively infected cells are detected by expression of both eGFP and dsRed, latently infected cells express only eGFP, and uninfected cells, or cells in which both reporters have been silenced, are eGFP^−^/dsRed^−^ (23). Jurkat-Tat cells were infected with VSV-G-pseudotyped mdHIV at an MOI of ∼0.1, a pool of the infected population (eGFP^+^), which included both latently and productively infected cells, was resorted for single cells into 96-well plates, and the clones were expanded over the following 2 months (Fig. 1B; see also Fig. S1 in the supplemental material). A total of 47 clonal lines derived and expanded from single cells were then reanalyzed by flow cytometry for expression of the LTR-linked dsRed and internal EIF1α-eGFP reporter in untreated cells and following stimulation with PMA (see Fig. S2 in the supplemental material). We found that after expansion over a period of several weeks, most of the parental lines (∼70%) produced a distinctive basal and PMA-induced dsRed (LTR) and eGFP (EIF1α) expression profile as measured by FACS in which expression of the internal EIF1α promoter appears to have been predominately, or at least partially, shut down in unstimulated cells but in which both the LTR-driven and internal promoters became induced by treatment with PMA after 24 h (e.g., clone 90 [Fig. 2A; see also Fig. S2 in the supplemental material]). A smaller proportion of the parental lines (∼15%) had retained complete expression of EIF1α-eGFP following expansion, but only some of these produced induction of LTR-dsRed in response to PMA (e.g., clone 11 [Fig. 2B]), while others did not induce dsRed expression (line 89 [Fig. 2C]). Additionally, ∼15% of the parental lines showed constitutive expression of both reporters in the majority of cells after clonal expansion which was only moderately affected by treatment with PMA (e.g., clonal line 46 [Fig. 2D]). The latter group likely represents cells that had established productive replication immediately upon infection and integration of the provirus (19, 23).

**FIG 3:**
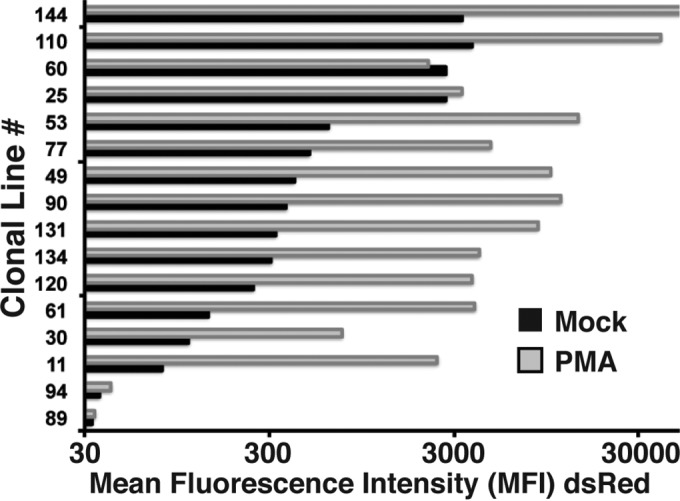
Mean fluorescence intensity (MFI) of 5′ LTR-dsRed expression for the indicated cloned cell lines was determined in untreated cells (mock) or cells treated with PMA for 24 h.

From these efforts, we selected 16 parental lines that produced a broad variety of PMA-induced expression of dsRed for further analysis, for which we calculated a mean fluorescence intensity (MFI) of LTR-dsRed expression produced in both untreated cells and cells treated with PMA for 24 h. As shown in Fig. 3, our analysis revealed that most of the parental lines produced significant induction of LTR expression in response to PMA, apart from parental lines with very low basal activity (e.g., lines 94 and 89) and lines with constitutive LTR expression (e.g., lines 60 and 25).

**FIG 4:**
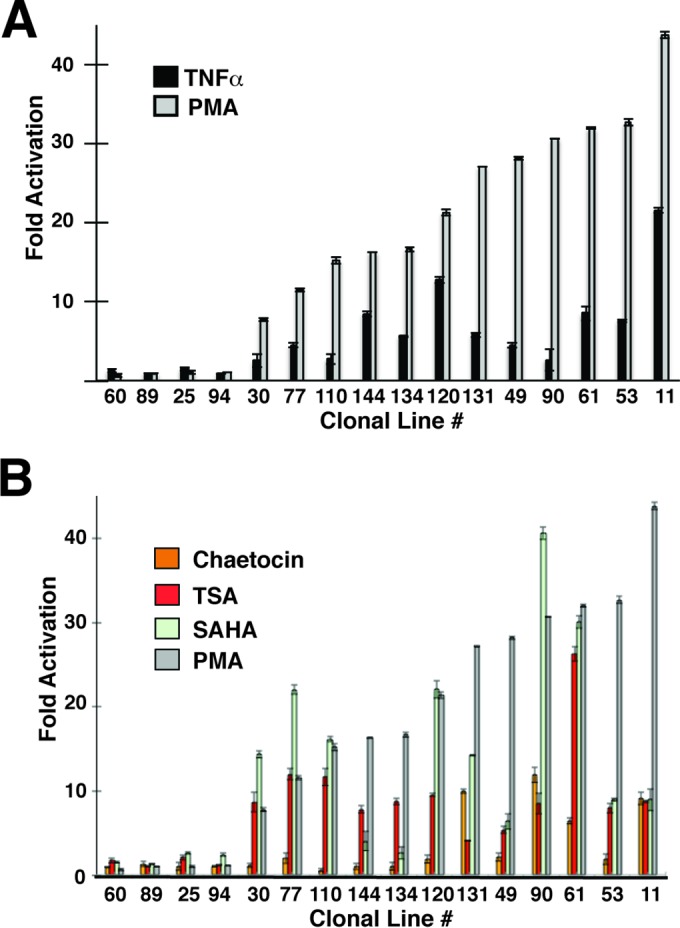
Response of LTR expression to signaling agonists and chromatin-modifying compounds. (A) The clonal cell lines were left untreated or treated for 24 h with PMA or TNF-α. Expression of dsRed from the 5′ LTR was measured by FACS analysis. Results are presented as fold activation of the MFI relative to that in the untreated samples. (B) Clonal cell lines were left untreated or treated 24 h with PMA, SAHA, chaetocin, or TSA. Expression of dsRed from the 5′ LTR was measured by FACS analysis. Results are presented as fold activation of MFI relative to that in the untreated samples.

We also compared responsiveness of the 16 parental cell lines to the effect of tumor necrosis factor alpha (TNF-α) and found that the response roughly correlated for the two agonists in many of the lines but not for lines 131, 49, 90, 61, and 53, which produced a proportionately lower response to TNF-α than to PMA (Fig. 4A; see also Fig. S3A in the supplemental material). Similarly, we found variable responses of LTR-eGFP expression in the parental lines to treatment with the HDAC inhibitors trichostatin A (TSA) and suberanilohydroxamic acid (SAHA) and the histone methyl transferase inhibitor (HMTI) chaetocin. For many of the lines, responsiveness to the HDAC inhibitors was approximately similar, with the exception of parental line 90, which showed significantly stronger induction of LTR expression by SAHA than by TSA (Fig. 4B). In general however, there was no correlation between the responsiveness of the mdHIV LTR to PMA or TNF-α with either of the HDAC inhibitors among the lines (see Fig. S3B and C). Responsiveness of LTR expression among the parental lines treated with chaetocin was typically less than with HDAC inhibitor-treated cells, which showed >5-fold induction only in lines 131, 90, 61, and 11 (Fig. 4B). Again, we did not observe a correlation between responsiveness of LTR expression to chaetocin and PMA or TNF-α (see Fig. S3D). These observations suggest that different integrations of HIV-1 provirus produce distinct phenotypes with respect to basal expression of the 5′ LTR, basal expression from the colinked EIF1α promoter, and responsiveness to T cell signaling agonists and chromatin-modifying compounds.

We confirmed that with the exception of two clones (60 and 25), the parental cell lines that produce these distinctive phenotypes represent unique single integration sites of proviral genomes, by determining the sequence of chromosomal DNA flanking the LTR for 12 of the lines (see Fig. S4 in the supplemental material). Consistent with previous studies (33, 34), we found that most of the parental lines had provirus integrated within introns of defined genes, while only 3 of the lines possessed integrations in intergenic regions (Table 2). Analysis of clones 60 and 25 revealed multiple sites of integration, and we believe that these reflect multiple clones within these expanded cultures, rather than multiple integrations in the same parental line, because further sorting experiments confirmed that they gave rise to subclones with distinct HIV phenotypes (see Fig. S5 in the supplemental material). Taken together, these results suggest that HIV provirus expression phenotype is strongly influenced by the site of chromosomal integration.

**TABLE 2:** Summary of HIV provirus integration sites

Line^*a*^	Chromosome	Coordinate^*b*^	Gene	Orientation^*d*^	Location	Description
11	19	1216102	*SKT11*	+	Intron	Serine/threonine kinase 11
25a	5	34806541	*RAI14*	−	Intron	*Homo sapiens* retinoic acid-induced 14 transcript variant 4
25b	12	57464390	*TMEM194A*	−	Intron	Transmembrane EM protein
30	13	84470733	NA^*e*^	NA	IG^*c*^	
49	8	21019060	NA	NA	IG	
77	11	66103261	*PACS1*	+	Intron	Human phosphofurin acidic cluster sorting protein 1
60a	1	150810825	*ARNT*	+	Intron	Aryl hydrocarbon receptor nuclear translocator
60b	22	38888776	*DDX17*	−	Intron	DEAD (Asp-Glu-Ala-Asp) box polypeptide 17
60c	7	97257666	*GPR63*	+	Intron	G protein-coupled receptor 63
89	1	28759261	*YTHDF2*	+	Intron	Human YTH domain family, member 2
94	4	496315	*ZNF721*	−	Intron	Zinc finger protein 721
110	11	13422727	*BTBD10*	−	Intron	BTB (POZ) domain containing 10
131	10	129738981	*MGMT*	+	Intron	*O*-6-Methylguanine-DNA methyltransferase
134	1	229721616	NA	NA	IG	
144	17	51078650	*SPAG9*	−	Intron	Sperm-associated antigen 9

a Clonal cell line number.

b Nucleotide coordinate of provirus integration site.

c IG, intergenic region.

d Provirus orientation relative to gene transcription.

e NA, not applicable.

### Parental expression profiles can be reproduced from subclones of cells with varied promoter activities.

**FIG 5:**
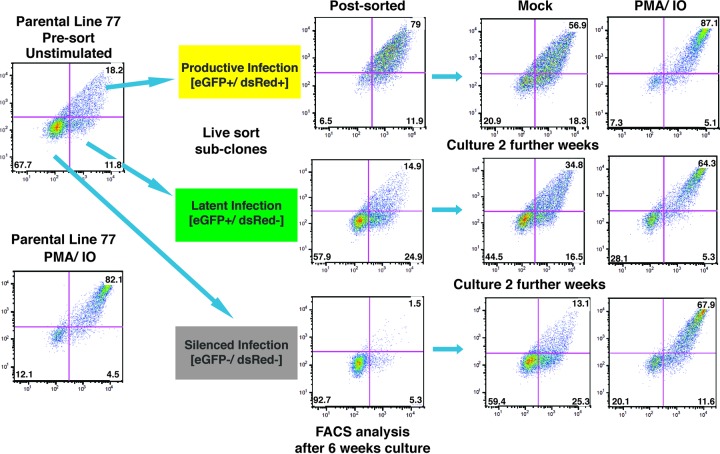
Analysis of provirus expression in parental line 77 subclones. Untreated parental line 77 (top left) or cells treated for 4 h with PMA and ionomycin (PMA/IO, bottom left) were analyzed by FACS for expression of dsRed (*y* axis) or eGFP (*x* axis). Single cell subclones of parental line 77 expressing both LTR-dsRed and EIF1α-GFP (productive infection), expressing only EIF1α-GFP (latent infection), or in which both reporters have been silenced (silenced infection) were isolated by live sorting and expanded in culture for 6 weeks; samples of the subcloned populations were reanalyzed by FACS (post-sorted). Subclones were cultured a further 2 weeks, and untreated cells (mock) or cells treated with PMA and ionomycin for 24 h (PMA/IO) were reanalyzed by FACS.

Each of the parental lines harboring mdHIV reporter provirus produced distinct scatter profiles of LTR-dsRed and EIF1α-eGFP expression, as determined by flow cytometry, which likely reflects the extent of transcriptional noise generated by the two promoters. Because most of the lines produce wide variations in expression, from completely repressed to active for both promoters, we assessed whether these variations in expression would be maintained over multiple generations of cell division. For this purpose, we live sorted five of the parental cell lines for which we had identified integration sites (parental lines 49, 61, 77, 90, and 144) for single cells that represented latently infected (eGFP^+^ dsRed^−^) or productively infected (eGFP^+^ dsRed^+^) cells, in addition to cells in which both reporters had become repressed or silenced (eGFP^−^ dsRed^−^) (see Fig. S1 in the supplemental material). Although we obtained subclones from resorting each of the parental lines, we recovered subclones representing all three expression patterns only for parental lines 77 and 90 (Table 3). Subclones generated from this analysis were expanded in culture for 6 weeks and then reanalyzed by FACS to examine expression of eGFP and dsRed. We selected parental line 77 for further detailed analysis because we isolated multiple replicates for each of the HIV expression phenotypes (latently infected, productively infected, and silenced) (Fig. 5 and Table 3). We found that after 6 weeks of culture, each of the expanded subclones from the three sorted populations contained cells where both reporters were silenced (Fig. 5, Post-sorted). Additionally, following a further 2 weeks in culture, each of the subclones that were initially sorted for their different eGFP and dsRed expression profiles showed a gradual progression toward recapitulation of the parental cell line expression patterns for both reporters (Fig. 5, Mock). Interestingly, stimulation of the subcloned cell cultures with PMA and ionomycin caused redistribution of all three populations toward an identical pattern of eGFP and dsRed expression that was produced by the parental presorted clone 77 line (Fig. 5, PMA/IO). We performed this analysis for each of the subclones produced from parental line 77 (Table 3) and obtained identical results for clones from each of the three sorted populations (see Fig. S6 in the supplemental material), indicating that capability to recapitulate the parental expression profile must be genetically determined rather than caused by selective adaptation or selection during cell culture. These results suggest that the stochastic expression and induction patterns of integrated HIV provirus are determined by the chromatin landscape at the site of chromosomal integration.

**TABLE 3:** Summary of subclones isolated with distinct provirus expression profiles

Parental line	No. of subclones generated from parental lines with the indicated provirus expression profile		
	Silenced infection, GFP^−^ dsRed^−^^*a*^	Latent infection, GFP^+^ dsRed^−^^*b*^	Productive infection, GFP^+^ dsRed^+^^*c*^
49	3	2	0
61	3	0	0
77	5	5	3
90	3	3	1
144	0	1	1

a Number of subclones isolated in which both LTR-dsRed and the internal EIF1α promoter had been silenced.

b Subclones isolated with silenced LTR-dsRed expression.

c Subclones with active LTR-dsRed and EIF-1α-GFP expression.

**FIG 6:**
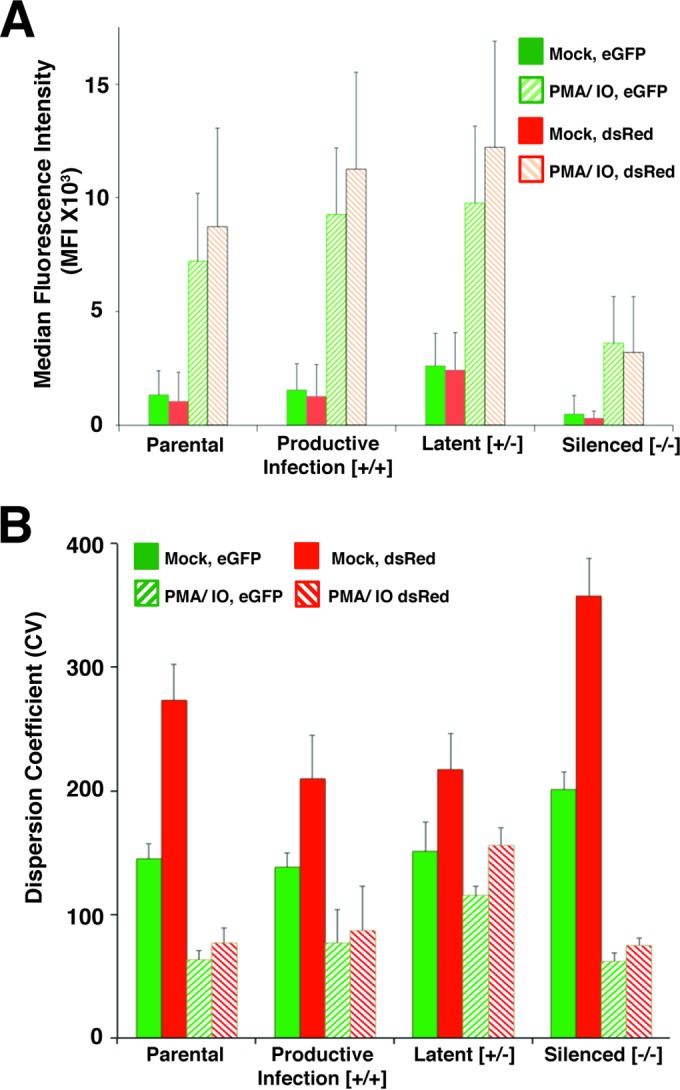
Quantitative analysis of provirus reporter gene expression for subclones of parental line 77. MFI (A) or CV (B) for EIF1α-eGFP and HIV 5′ LTR-dsRed expression were determined for parental line 77, and subclones were isolated that expressed both dsRed and eGFP (productive infection [+/+]), expressed eGFP but not dsRed (latent infection [+/−]), or had both reporters silenced ([−/−]). FACS analysis was performed on populations of untreated cells (mock) or cells treated with PMA-IO at 4 weeks after isolation of the subclones.

We also performed quantitative analysis of LTR-dsRed and EIF1α-eGFP expression in the subclones and compared the results to those produced by parental line 77. For this, we determined the mean fluorescence intensity (MFI) and dispersion coefficient of variation (CV) for both reporters, at 6 weeks after isolation of the subclones produced from the productively and latently infected or silenced populations, and compared these to values for the parental line (Fig. 6). By this measure, the MFI is directly proportional to the reporter expression level, while the CV represents diversity of the cell population, where a low value indicates a uniform expression level among the population and a high value reflects a more diverse or heterogeneous level of reporter expression. In this analysis, we found that subclones generated from productive, latently infected, and silenced cells within the parental line 77 population eventually repopulate their respective expression patterns upon prolonged culture, as measured by overall expression for each of the reporters (MFI [Fig. 6A]) and heterogeneity of expression (CV [Fig. 6B]). However, consistent with the qualitative analysis described above (Fig. 5; see also Fig. S6 in the supplemental material), clones generated from the silenced population of line 77 produced a slower transition toward repopulating the productive and latent expression patterns, particularly with respect to overall expression of the reporters (Fig. 6A). In contrast, cultures derived from the latently and productively infected parental line 77 populations reverted more rapidly to an MFI and CV that resembled those of the parental line (Fig. 5). This indicated that there may be significantly more mechanistic pressure to maintain the early latent HIV provirus in unstimulated cells than there is to revert to latency from productive infections. We discuss implications of this observation in more detail below.

### Recapitulation of the parental provirus expression phenotype is a typical property of different HIV integrants.

**FIG 7:**
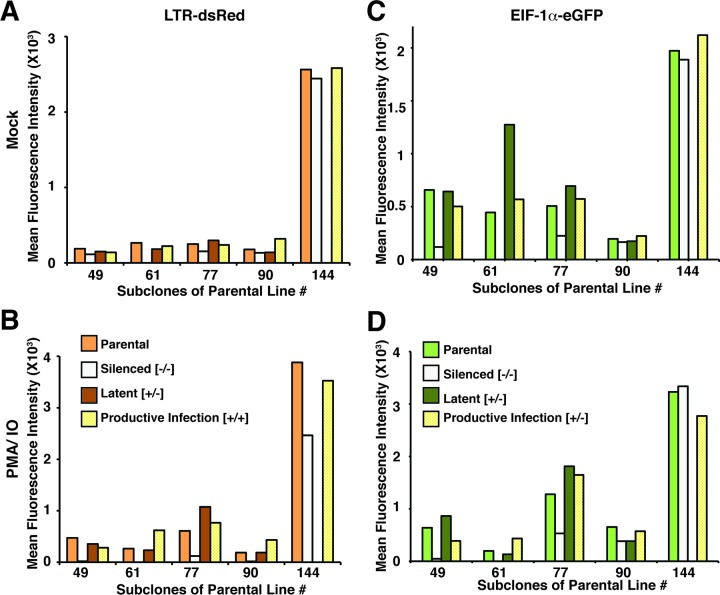
Analysis of MFI of HIV 5′ LTR-dsRed (A and B) and EIF1α-eGFP (C and D) expression in sublcones of parental lines 49, 61, 77, 90, and 144. The parental cloned line (parental) or subclones isolated by FACS for expression of both eGFP and dsRed (productive infection), subclones expressing only eGFP (latent), or subclones in which both reporters had been silenced (silenced) were analyzed 4 weeks postisolation. Cell populations were left untreated (mock [A and C]) or treated with PMA-IO for 24 h (B and D) prior to analysis by FACS.

**FIG 8:**
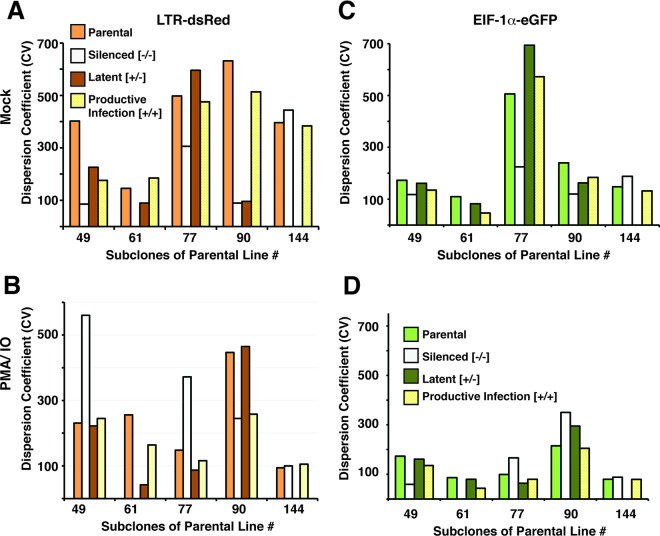
Analysis of the CV for HIV 5′ LTR-dsRed (A and B) and EIF1α-eGFP (C and D) expression in sublcones of parental lines 49, 61, 77, 90, and 144. The parental cloned line (parental) or subclones isolated by FACS for expression of both eGFP and dsRed (productive infection), subclones expressing only eGFP (latent), or subclones in which both reporters had been silenced (silenced) were analyzed 4 weeks postisolation. Cell populations were left untreated (mock [A and C]) or treated with PMA-IO for 24 h (B and D) prior to analysis by FACS.

To determine whether the effects we observed with parental line 77 are representative of HIV provirus phenotypes in general, we analyzed expression characteristics for subclones of four additional parental cell lines (49, 61, 90, and 144) in parallel with further analysis of line 77. For this experiment, we expanded a single clone, from the latent (eGFP^+^/dsRed^−^), productive (eGFP^+^/dsRed^+^), and silenced (eGFP^−^/dsRed^−^) populations for each of the cell lines, where available (Table 3), for a period of 6 weeks. We then examined the MFI and dispersion CV of the expanded subclones in parallel with their parental lines in both untreated and PMA-treated cultures. We found that consistent with the results shown above, in general the majority of the subcloned cultures produced an MFI and CV for LTR-dsRed and EIF1α-eGFP expression that were comparable to those of the parental cell line in both untreated and treated cells (Fig. 7 and 8). Thus, for example, all three untreated subclones produced from parental line 90 generated an approximately equivalent MFI for LTR-dsRed and EIF1α-eGFP (Fig. 7A and C), and also for the PMA-IO-stimulated cultures (Fig. 7B and D). Additionally, the dispersion coefficient for LTR-dsRed expression of the three subclones generated from parental line 90 were also approximately the same for both PMA-IO-stimulated and untreated cells (Fig. 8A and B, respectively). However, in this case, there was some variability in the dispersion coefficient for EIF1α-eGFP expression between several of the clones (latent, eGFP^+^ dsRed^−^) and (silenced, eGFP^−^ dsRed^−^), particularly for the untreated cultures (Fig. 8C and D, clone 90). Nevertheless, by this analysis, the majority of subclones from the four additional lines generally reproduced the parental expression phenotype for both LTR-dsRed and EIF1α-eGFP after 8 weeks of culture. These observations support a view that the chromosomal landscape surrounding the site of integration strongly dictates expression phenotype of integrated HIV provirus.

### Variation in Tat activity cannot account for differences in early latent HIV provirus phenotypes.

**FIG 9:**
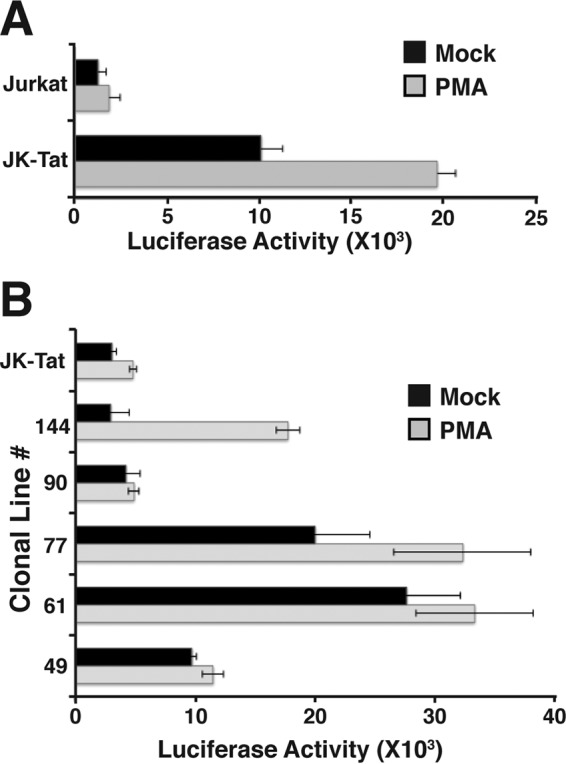
Analysis of Tat/TAR-dependent HIV LTR expression in parental lines 49, 61, 77, 90, and 144. (A) Jurkat or Jurkat-Tat (JK-Tat) cell lines were transfected with an HIV-1 LTR (TAR^+^)-luciferase reporter plasmid and incubated for 48 h, and luciferase activity was measured 24 h later in untreated samples (mock) or cells treated with PMA. Results were normalized for transfection efficiency by expression of β-galactosidase from a cotransfected RSV β-Gal expression plasmid. (B) JK-Tat or the parental lines as indicated were transfected with the LTR-TAR-reporter plasmid, and luciferase activity was measured in untreated (mock) or PMA-treated cells as for panel A.

Since we have been unable to reliably generate stable lines infected with dual-reporter HIV that expresses all of the viral accessory gene products, we used a mini-dual-reporter HIV (mdHIV) (Fig. 1A) which does not express viral accessory molecules, notably Tat. This necessitated the use of Jurkat-Tat cells, which constitutively express Tat from an integrated transgene. Consequently, we wondered whether differences in the HIV provirus phenotypes could be attributed to altered Tat activity in these cells. Because Tat function is modulated by multiple posttranslational modifications (35, 36), we assessed potential differences in Tat function between the cell lines by measuring its effect on TAR-dependent expression from the HIV LTR. For this purpose, we transiently transfected each of the lines, and the parental Jurkat-Tat line, with an HIV-1 LTR-luciferase (TAR^+^) reporter and measured expression of luciferase in untreated and PMA-stimulated cells. Transfection efficiency was normalized by cotransfection of an RSV β-galactosidase internal control reporter gene plasmid. This plasmid is responsive to Tat function, as we observed significantly higher luciferase expression in Jurkat-Tat cells than in Jurkat cells (Fig. 9A). In transfections of the cloned parental lines, we did find some variability in LTR-directed luciferase expression, but most of the lines produced higher levels of luciferase expression than did the uninfected Jurkat-Tat line (Fig. 9B). We do not believe that the differences in transient LTR activity can account for production of the various HIV provirus phenotypes we observed, because there was no correlation between provirus expression phenotype and Tat activity in the parental cell lines. These findings suggest that Tat activity is already saturated in the parental Jurkat-Tat line, and differences observed in the transient-transfection assays might reflect subtle changes in Tat expression produced over months of culture after live sorting of lines. We note that the Tat is expressed from a BK virus vector in the Jurkat-Tat line and is maintained by coexpression of a Neo^r^ (G418 resistance) marker (37). Consequently, it is likely that long periods of culture in medium containing G418 may have selected for amplified Tat expression in at least some of the lines relative to that in the original Jurkat-Tat line. Nevertheless, since the identical basal level and PMA-induced expression profile were observed in all of the parental lines and subclones after many weeks of culture, variations in expression of Tat cannot explain differences in phenotypes produced by the provirus lines.

### Early latent infections are established in Jurkat-Tat cells by the RGH reporter virus.

**FIG 10:**
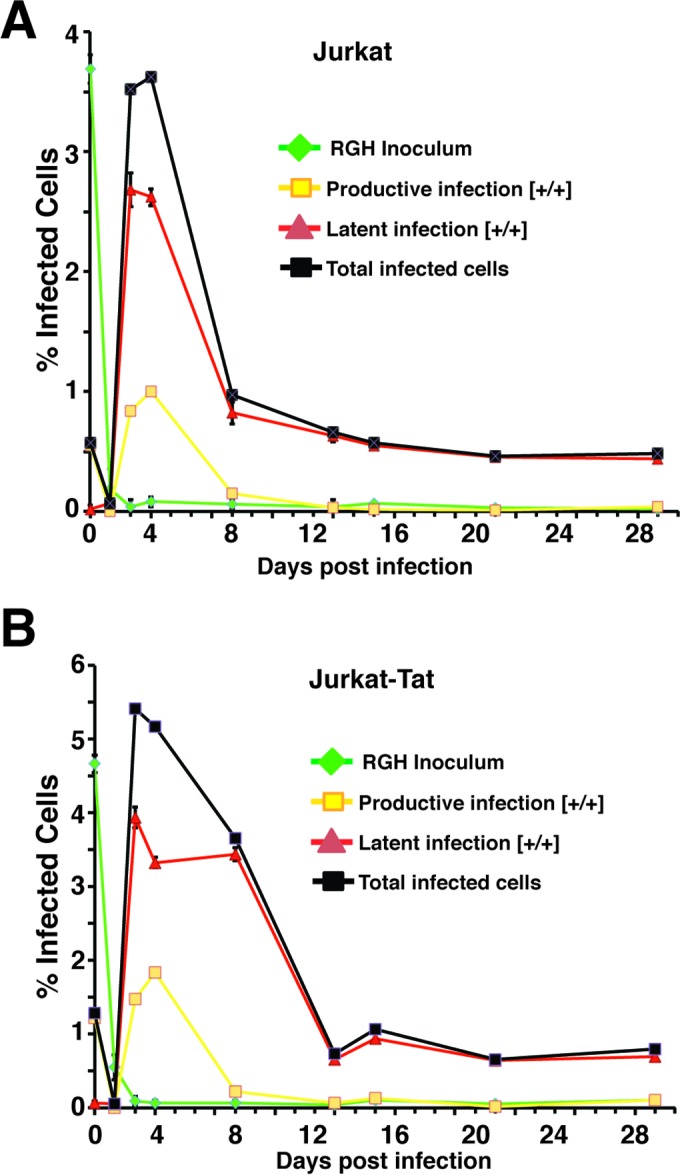
Full-length HIV reporter virus (RGH) expressing all accessory factors, apart from Nef, establishes immediate latency in Jurkat T cells constitutively expressing Tat. The red-green HIV reporter virus (RGH) expresses eGFP from the 5′ LTR and mCherry from an internal PGK promoter, inserted within *Nef* (22), but otherwise expresses all of the HIV gene products (see Fig. S6 in the supplemental material). Jurkat cells (A), or Jurkat-Tat cells (B) were infected with VSV-G-pseudotyped RGH virus, and cells were analyzed for expression of eGFP and mCherry by FACS at the indicated times. Inoculated cells are initially detected by eGFP, which is carried by the RGH virion (inoculum HIV). Productively infected cells are subsequently detected by expression of both eGFP and mCherry ([+/+]), while latently infected cells produce mCherry from the internal PGK promoter but not eGFP ([+/−]). The proportions of total infected cells are indicated.

We note that with the mini-dual-HIV reporter virus, we observed approximately the same proportion of early latent cells produced upon infection of cells that constitutively express Tat (23) as in previous experiments with a dual-reporter vector (red-green HIV [RGH]) which expresses all of the viral gene products and accessory factors, apart from Nef (19, 22). From this result, we wondered whether the absence of the additional viral gene products enables the minivirus to establish early latent infections, despite the effect of constitutive Tat expression. To examine this, we used the RGH virus (see Fig. S7 in the supplemental material), in which GFP is expressed from the 5′ LTR and mCherry is produced from an internal constitutive PGK promoter (19), to infect both Jurkat and Jurkat-Tat cells. Note that with this virus, during packaging GFP becomes incorporated into the virion, and consequently, all newly infected cells are initially GFP^+^ for several hours until this signal decays, after which the GFP signal is dependent upon expression from the LTR (19). In these experiments, we found no difference in the proportion of cells that produced early latent infections, typified by expression of mCherry but not GFP (mCherry^+^ GFP^−^) (Fig. 10), or those which produced productive infections (mCherry^+^ GFP^+^) for several weeks postinfection. However, the proportion of total infected cells in both cultures decreased sharply 2 weeks postinfection, which likely represented cytopathic effects of viral gene products. Nine days postinfection, cells harboring productive infections (mCherry^+^ GFP^+^) were eliminated, or virus expression had reverted to latency in both cultures. Additionally, the proportion of latently infected cells (mCherry^+^ GFP^−^) stabilized at a constant level by day 14 and persisted for at least two further weeks. These results indicate that HIV is able to establish early latency immediately upon infection and maintain a stable population of these cells, even in cells that constitutively express Tat. This implies that mechanisms required for production of this mode of latency may be different than those previously described for HIV that productively replicates upon infection (38–40).

## DISCUSSION

There is currently a consensus that a cure for HIV disease will require a means of purging, or effectively eliminating, latently infected cells, which are unaffected by current therapies and can act as a reservoir for viral rebound in HIV-infected individuals (1). One strategy that has attracted considerable attention is known as shock and kill, where proposed therapeutic intervention would induce provirus gene expression to allow clearance of the latently infected cells by the host immune response or virus-induced apoptosis (41). Much of the effort toward this strategy to date has focused on HDAC inhibitors, several of which were shown to cause induction of latent HIV provirus in culture (42). Clinical trials with HDAC inhibitors have shown capability of inducing HIV expression in patients on antiretroviral therapy, but overall, the treatment did not significantly affect the latently infected population (28), and replication-competent virus could still be recovered from stimulated T cells isolated from patient samples following treatment. Resilience of the latent HIV population to treatment with HDAC inhibitors has been attributed to the possibility that provirus integrated at different chromosomal locations may be differentially responsive to the effects of chromatin-modifying drugs. This is supported by observations indicating differential responses to a variety of T cell signaling agonists and epigenetic modifying compounds on various established models for HIV latency.

In this study, we have examined the possibility that the site of chromosomal integration can affect the phenotypic expression of HIV provirus, by isolating multiple clonal cell lines harboring a single-copy integrant of a mini-dual-fluorescence reporter HIV (mdHIV) (23). Cloned provirus-bearing lines were isolated by FACS following infection with mdHIV, and a collection of these were selected for further analysis, displaying a variety of basal and PMA-induced reporter gene expression profiles. As expected, each of the parental lines harbored a unique single copy of the mdHIV provirus, mostly within introns of defined genes, as well as several within intergenic regions. While we observed a correlation between responsiveness to PMA and TNF-α for some lines, overall, there was no correlation between responsiveness of clones to PMA or TNF-α and various chromatin-modifying agents. The finding that some lines that are highly responsive to PMA but disproportionately less so to TNF-α is surprising, considering that these agonists are thought to cause activation of an overlapping set of LTR-bound transcriptional activators (8). In contrast, considering results from clinical trials, it might not be surprising to find that some of the provirus lines were relatively unresponsive to TSA or SAHA, despite being highly responsive to PMA (lines 134, 49, and 11 [Fig. 3]). On the other hand, several of the lines that showed significant response to SAHA were less responsive to PMA (for example, lines 30 and 77), suggesting that some proviruses are particularly sensitive to chromatin remodeling and less dependent upon activation by transactivators downstream of T cell signaling. Furthermore, although we only examined the effect of chaetocin in this study, it appears that only a small proportion of proviruses are sensitive to effects of histone methyl transferase inhibitors (HMTIs). HMTIs are known to produce synergistic responses with HDAC inhibitors in a variety of provirus HIV model systems (12, 13, 31, 43), and although this study has not examined this phenomenon directly, we suppose that a significant number of the latently infected clones might show a similar synergistic response.

Our results have important implications for the proposed shock-and-kill strategy to eliminate latently infected cells from patients. First, it appears that provirus integrated at different chromosomal locations will require different shock treatments to produce reactivation. For example, provirus integrated at some locations may be reactivated by HDAC inhibitors alone, while others may require demethylation at histone H3K9 and/or H3K27 (44, 45). Still other proviruses may require stimulation of one or more signaling pathway in addition to or instead of alterations in chromatin modification (46). It will obviously be important to establish which mechanism(s) contributes to silencing of latent populations in clinically relevant populations, as this will define which potential shock molecules might be effective. This supports a view that an effective shock-and-kill regimen will require a combination of therapies that target the latent reservoir by different mechanisms to stimulate a broader spectrum of the provirus population (47). On the other hand, our data indicate that many integration sites produce significant basal transcriptional “noise,” as demonstrated by expanding clones isolated as both latent and productively infected cells. This indicates that provirus at some sites may not require treatment, as cells with integrations at these locations may eventually shock themselves through noisy basal expression. In any case, it seems clear that a better understanding of the integrations site profile of latent provirus in patients on therapy will be important toward development of therapies to eliminate this population. Finally, an additional consideration is that most studies focused on potential shock-and-kill strategies have been performed with T cells, and very little is known regarding corresponding responses and the effect of provirus integration sites in monocytes/macrophages, which represent an additional important reservoir for latent provirus in patients (48).

In addition to differential responses to T cell signaling and chromatin-modifying agonists, each of our cloned parental lines produced distinctive basal expression patterns for both the LTR and internal EIF1α promoters. A previous study has shown that basal expression of an HIV LTR-GFP reporter varies by as much as 75% between individual clones, depending on the site of integration (49). With the dual reporter mdHIV construct, we were able to detect infection independently of viral gene expression, and consequently, we found a significantly larger difference in basal activity, with as much as a 100-fold difference between the lowest to highest MFI for basal dsRed (LTR) expression among the clones. Additionally, although infected cells were initially selected for expression of the internal EIF1α-eGFP reporter, most of the cloned parental lines lose expression of eGFP following 3 weeks of expansion and reanalysis by FACS (see Fig. S2 in the supplemental material). This observation suggests that the integrated provirus must cause silencing of the otherwise constitutive colinked PGK-EIF1α, likely by mechanisms involving spreading of repressive epigenetic marks from the HIV LTR (8). A few of the latently infected parental lines harbored provirus that showed very low basal levels of LTR expression and did not respond to any of the signaling or chromatin-modifying agonists (lines 89 and 94). It might be expected that this type of HIV expression phenotype would be produced by proviral integration into a heterochromatic region, but curiously, clone 94 has an integration of the mdHIV reporter virus within an intron of the *ZNF721* gene, although in the reverse orientation (Table 2). It is possible that this provirus is rendered nonresponsive by a mechanism that may involve transcriptional interference with the cellular gene regulatory mechanisms.

Most of the cloned parental lines generate significantly diverse basal expression for both the LTR and internal EIF1α promoters, likely representing transcriptional noise generated by a stochastic signaling environment in unstimulated cells (50, 51). We found that subclones of the lines, which were sorted by FACS for expression patterns representing latent (dsRed^−^ eGFP^+^), productively replicating (dsRed^+^ eGFP^+^), or silenced provirus (dsRed^−^ eGFP^−^), recapitulated the identical full basal expression profile of the parental line within several weeks of subculture. We have observed this effect with at least five of the parental lines, each of which produces a unique basal HIV expression phenotype, and this indicates that transcription factors that bind the LTR in unstimulated cells must restore transcription levels to the original basal state, despite any potential influences from flanking regulatory regions at the site of HIV integration. Thus, while the site of integration can influence responsiveness to various signaling and chromatin-modifying agonists, HIV LTR function seems to dominate over the flanking environment for basal expression. It is somewhat surprising that cells isolated from the productively infected population (dsRed^+^ eGFP^+^) gradually revert to the parental basal phenotype, given that these cells constitutively express Tat. This finding implies that there must be mechanisms that can overcome the effect of Tat function to enable LTR silencing and reestablishment of latency. Such mechanisms might be similar, if not identical, to those involved in establishment of early latency in newly infected cells, a feature we discuss in more detail below. We found that in general, cells cloned from the productively infected (dsRed^+^ eGFP^+^) and latently infected (dsRed^−^ eGFP^+^) cultures revert back to the parental phenotype more rapidly than do cells from clones of the HIV-silenced population (dsRed^−^ eGFP^−^). A possible explanation for this finding is that the silenced provirus population, which has lost expression of both the LTR and internal EIF1α promoters, may have accumulated epigenetic marks that take longer to reverse in culture than the latently and productively infected populations. Taken together, these findings suggest that there is a strong propensity for HIV provirus to maintain a latent state in infected but unstimulated cells.

Several studies have demonstrated that the positive-feedback effect of Tat on viral transcription is critical for overcoming mechanisms that promote latency of HIV-1 provirus (18, 38, 52), and given these reports, it may be expected that cells which constitutively express Tat should not give rise to latently HIV-infected cells. However, we note that all previous studies examining the effect of Tat utilized an LTR-driven reporter as a sole source of detection of HIV infection. Using the mini-dual-reporter HIV, we found that ∼50% of infected cells produced latent provirus soon after infection in Jurkat-Tat cells, which constitutively express Tat protein. We have confirmed these findings by showing that a dual-reporter virus (RGH) which expresses all of the viral gene products except for Nef produces similar results. Our finding that early latency can be established despite constitutive expression of Tat supports a view that establishment of early latency may involve an independent pathway, separate from the gradual epigenetic silencing of productive virus infections toward proviral latency, as described previously (53). While YY1 protein seems to be an important determinant for establishment of early latency (23), the details of its function for this mode of latency have not yet been elucidated. YY1 can act as a transcriptional activator or repressor, is capable of binding both DNA and RNA, and can recruit a variety of epigenetic modifying complexes (54), and consequently, it is not clear how this factor may contribute to establishment of early latency.

A better understanding of the mechanisms that contribute to establishment and maintenance of the latent HIV reservoir is necessary for development of therapies to eradicate the latently infected population from patients with HIV disease. In this study, we have directly examined the role of HIV integration sites in expression phenotypes of provirus and found significant differences in responsiveness to T cell signaling agonists and chromatin-modifying compounds. Our results are consistent with the view that the latent HIV-infected cell population is not only very heterogeneous but also stable and that a combination of therapies will be necessary to eradicate latent HIV-infected cells from patients with HIV/AIDS disease.

## Supplementary Material

Supplemental material
