# Supplementary material for: HIV Provirus Stably Reproduces Parental Latent and Induced Transcription Phenotypes Regardless of the Chromosomal Integration Site
Source: J Virol. 2016 May 12;90(11):5302–14. doi: 10.1128/JVI.02842-15 (PMC4934743; doi:10.1128/JVI.02842-15)
Supplement: Supplemental material [file supp_90_11_5302__index.html]

HIV Provirus Stably Reproduces Parental Latent and Induced Transcription Phenotypes Regardless of the Chromosomal Integration Site — Supplemental material 

# HIV Provirus Stably Reproduces Parental Latent and Induced Transcription Phenotypes Regardless of the Chromosomal Integration Site

## Supplemental material

- Supplemental file 1 -

  Fig. 1 (Analysis of mdHIV provirus expression phenotypes in clonal cell lines.)

  Fig. 2 (FACS analysis of cloned parental lines.)

  Fig. 3 (Comparison of provirus responses to signaling agonists and chromatin-modifying compounds.)

  Fig. 4 (Identification of mdHIV integration sites by nested PCR.)

  Fig. 5 (FACS analysis of line 25 and 60 subclones following 6 weeks of culture.)

  Fig. 6 (FACS analysis of line 77 subclones following 6 weeks of culture.)

  Fig. 7 (Schematic representation of the PGK RGH reporter virus.)

  Supplemental figure legends.

  PDF, 2.0M
