# Supplementary material for: HIV Provirus Stably Reproduces Parental Latent and Induced Transcription Phenotypes Regardless of the Chromosomal Integration Site
Source: J Virol. 2016 May 12;90(11):5302–14. doi: 10.1128/JVI.02842-15 (PMC4934743; doi:10.1128/JVI.02842-15)
Supplement: Supplemental material [file JVI.02842-15_zjv999091662so1.pdf]

Supplementary Figure S1 – Analysis of mdHIV provirus expression phenotypes in clonal cell lines.

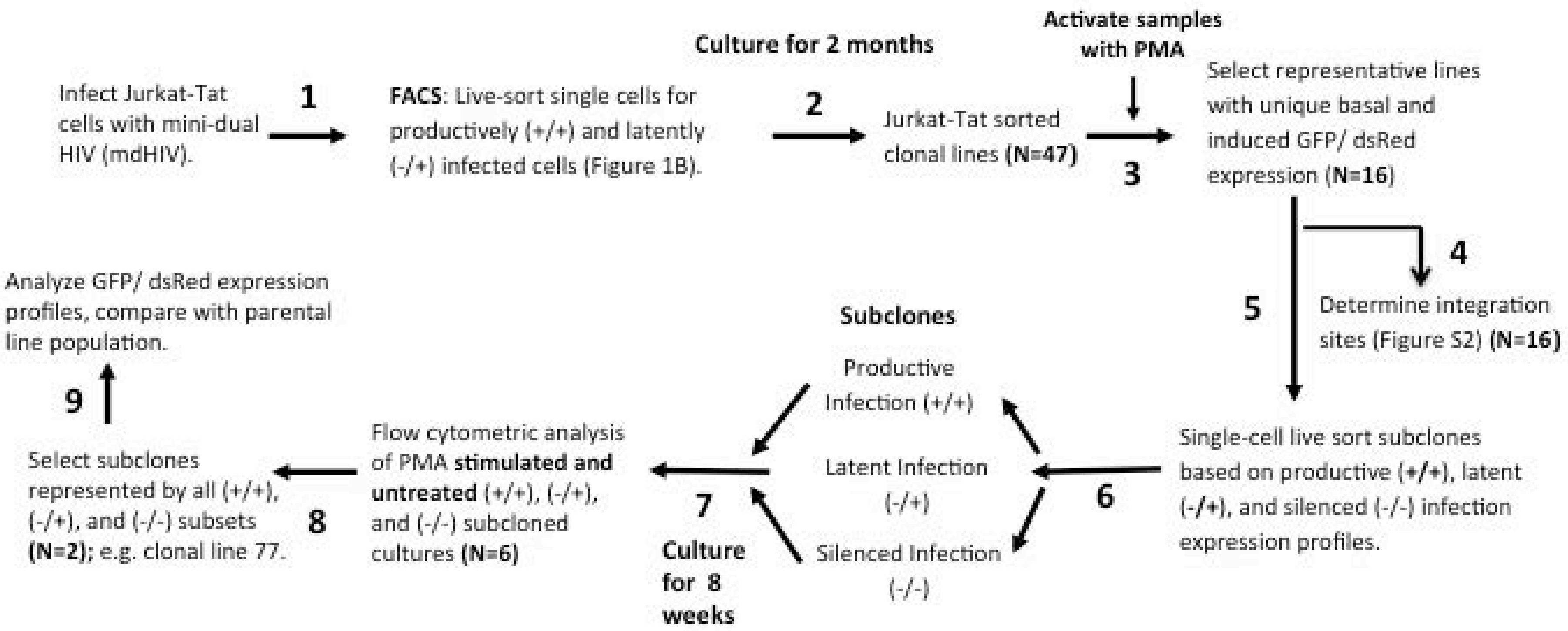

Supplementary Figure S2 – FACS analysis of cloned parental lines

Class A (Indicated in Figure 2)

#7 Untreated

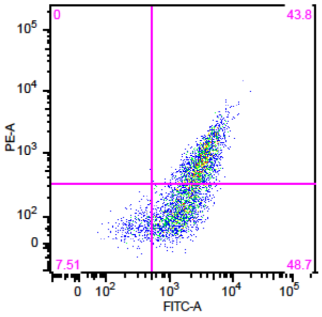

FSC-A, SSC-A subset
Specimen_001_7.fcs
Event Count: 3904

#7 PMA

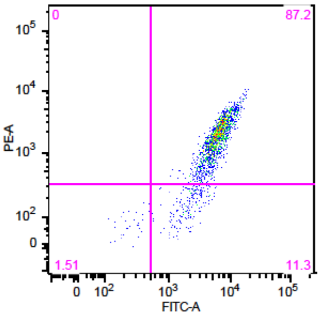

FSC-A, SSC-A subset
Specimen_001_7p.fcs
Event Count: 1788

#8 Untreated

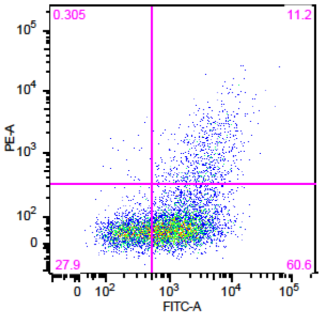

FSC-A, SSC-A subset
Specimen_001_8.fcs
Event Count: 6555

#8 PMA

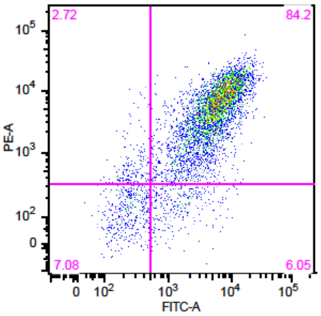

FSC-A, SSC-A subset
Specimen_001_8p.fcs
Event Count: 5554

#19 Untreated

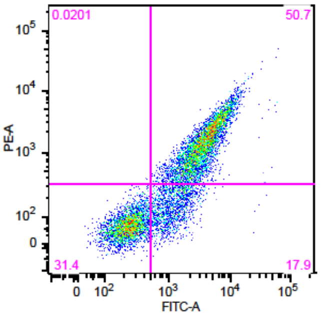

FSC-A, SSC-A subset
Specimen_001_19.fcs
Event Count: 9968

#19 PMA

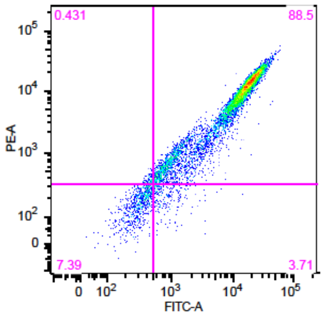

FSC-A, SSC-A subset
Specimen_001_19p.fcs
Event Count: 9969

Supplementary Figure S2 – FACS analysis of cloned parental lines

Class A (Indicated in Figure 2)

#24 Untreated

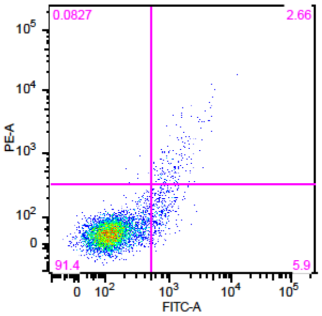

FSC-A, SSC-A subset
Specimen_001_24.fcs
Event Count: 6049

#24 PMA

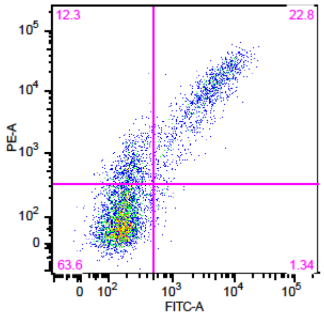

FSC-A, SSC-A subset
Specimen_001_24p.fcs
Event Count: 4996

#26 Untreated

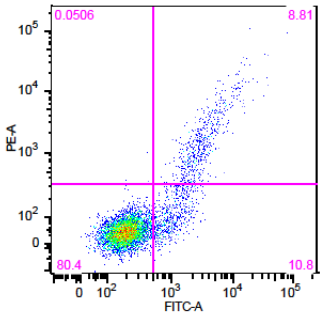

FSC-A, SSC-A subset
Specimen_001_26.fcs
Event Count: 5924

#26 PMA

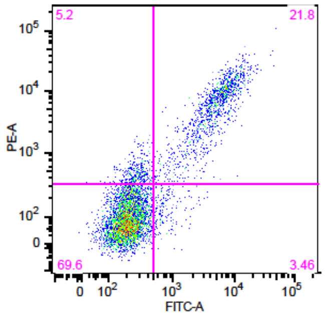

FSC-A, SSC-A subset
Specimen_001_26p.fcs
Event Count: 5524

#30 Untreated

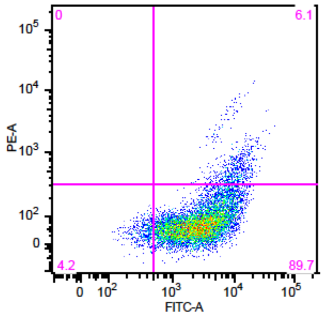

FSC-A, SSC-A subset
Specimen_001_30.fcs
Event Count: 9970

#30 PMA

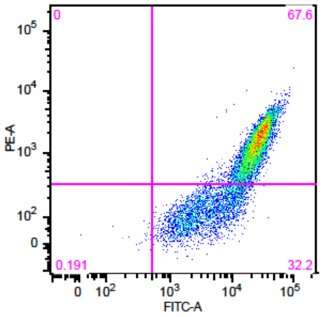

FSC-A, SSC-A subset
Specimen_001_30p.fcs
Event Count: 9928

Supplementary Figure S2 – FACS analysis of cloned parental lines

Class A (Indicated in Figure 2)

#32 Untreated

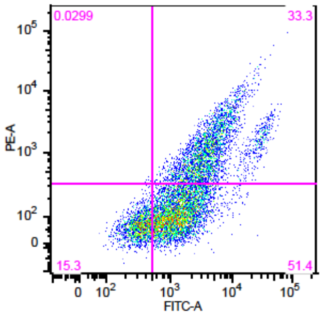

FSC-A, SSC-A subset
Specimen_001_32.fcs
Event Count: 10050

#32 PMA

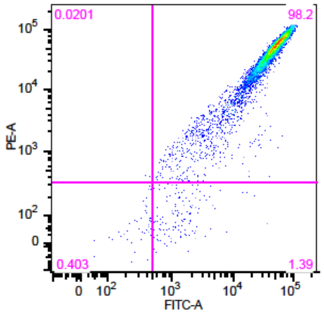

FSC-A, SSC-A subset
Specimen_001_32p.fcs
Event Count: 9937

#43 Untreated

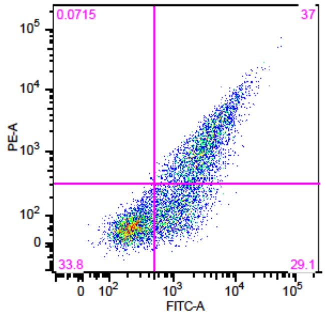

FSC-A, SSC-A subset
Specimen_001_43.fcs
Event Count: 6990

#43 PMA

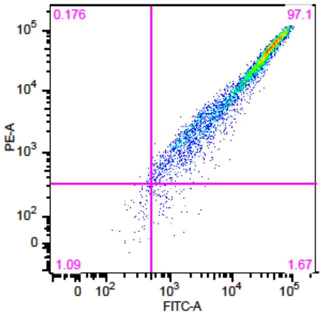

FSC-A, SSC-A subset
Specimen_001_43p.fcs
Event Count: 6241

#45 Untreated

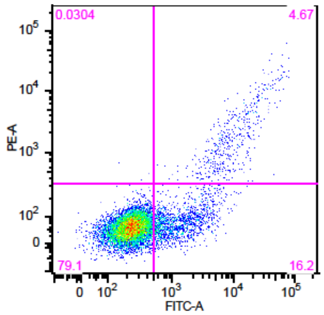

FSC-A, SSC-A subset
Specimen_001_45p.fcs
Event Count: 9868

#45 PMA

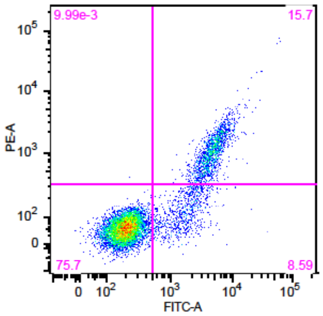

FSC-A, SSC-A subset
Specimen_001_45.fcs
Event Count: 10012

Supplementary Figure S2 – FACS analysis of cloned parental lines

Class A (Indicated in Figure 2)

#49 Untreated

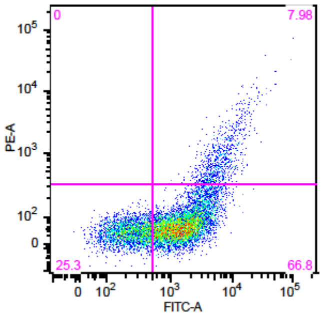

FSC-A, SSC-A subset
Specimen_001_49.fcs
Event Count: 9973

#49 PMA

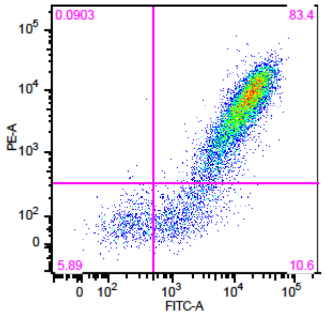

FSC-A, SSC-A subset
Specimen_001_49p.fcs
Event Count: 9968

#52 Untreated

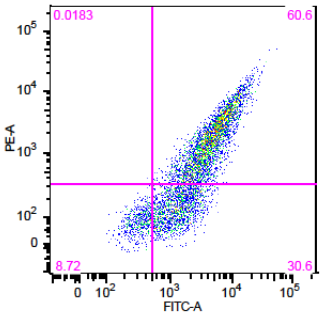

FSC-A, SSC-A subset
Specimen_001_52.fcs
Event Count: 5473

#52 PMA

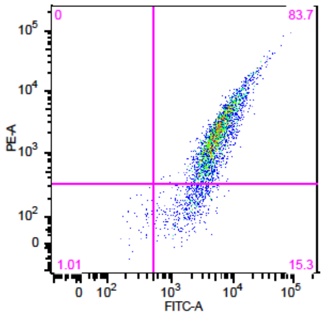

FSC-A, SSC-A subset
Specimen_001_52p.fcs
Event Count: 3579

#61 Untreated

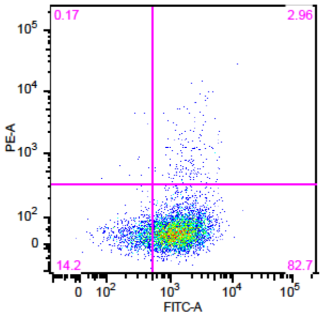

FSC-A, SSC-A subset
Specimen_001_61.fcs
Event Count: 5875

#61 PMA

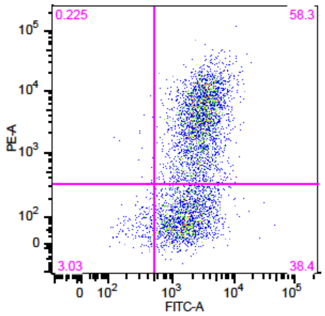

FSC-A, SSC-A subset
Specimen_001_61p.fcs
Event Count: 4881

Supplementary Figure S2 – FACS analysis of cloned parental lines

Class A (Indicated in Figure 2)

#66 Untreated

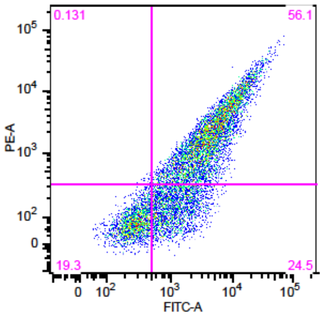

FSC-A, SSC-A subset
Specimen_001_66.fcs
Event Count: 9941

#66 PMA

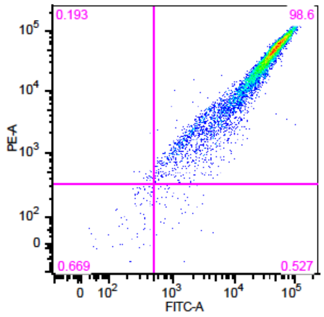

FSC-A, SSC-A subset
Specimen_001_66p.fcs
Event Count: 9861

#68 Untreated

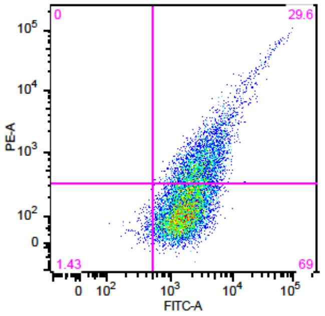

FSC-A, SSC-A subset
Specimen_001_68.fcs
Event Count: 9997

#68 PMA

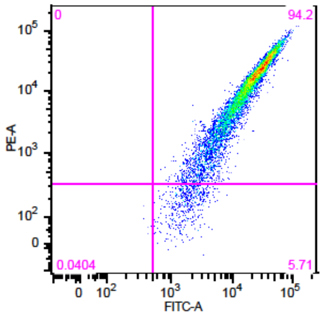

FSC-A, SSC-A subset
Specimen_001_68p.fcs
Event Count: 9889

#76 Untreated

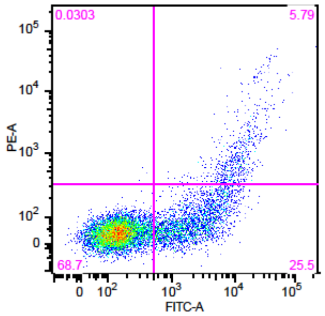

FSC-A, SSC-A subset
Specimen_001_76.fcs
Event Count: 9891

#76 PMA

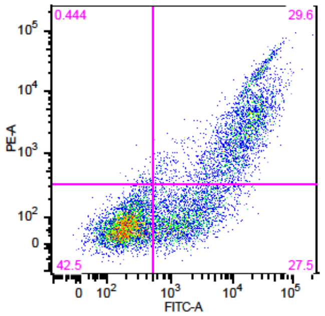

FSC-A, SSC-A subset
Specimen_001_76p.fcs
Event Count: 9914

Supplementary Figure S2 – FACS analysis of cloned parental lines

Class A (Indicated in Figure 2)

#77 Untreated

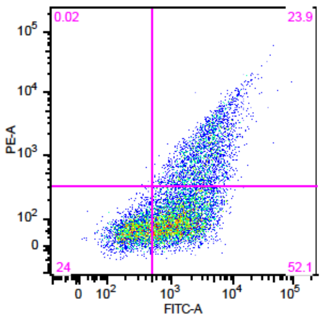

FSC-A, SSC-A subset
Specimen_001_77.fcs
Event Count: 9998

#77 PMA

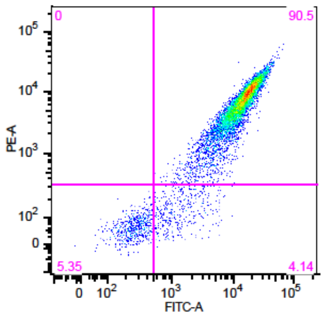

FSC-A, SSC-A subset
Specimen_001_77p.fcs
Event Count: 9932

#84 Untreated

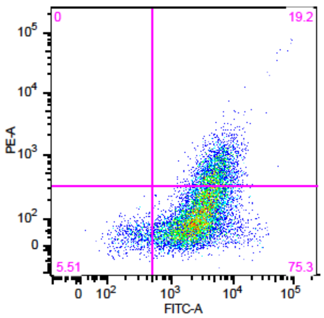

FSC-A, SSC-A subset
Specimen_001_84.fcs
Event Count: 9962

#84 PMA

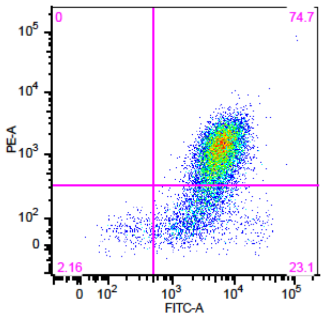

FSC-A, SSC-A subset
Specimen_001_84p.fcs
Event Count: 9973

#85 Untreated

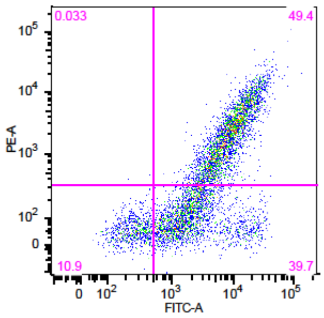

FSC-A, SSC-A subset
Specimen_001_85.fcs
Event Count: 6068

#85 PMA

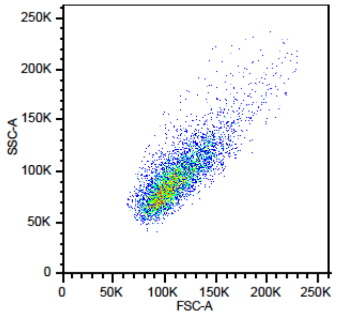

FSC-A, SSC-A subset
Specimen_001_85p.fcs
Event Count: 5246

Supplementary Figure S2 – FACS analysis of cloned parental lines

Class A (Indicated in Figure 2)

#86 Untreated

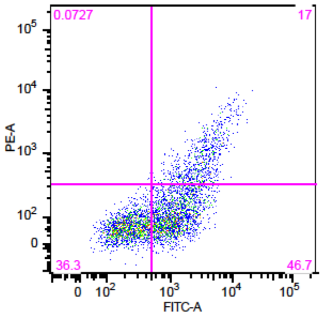

FSC-A, SSC-A subset
Specimen_001_86.fcs
Event Count: 4125

#86 PMA

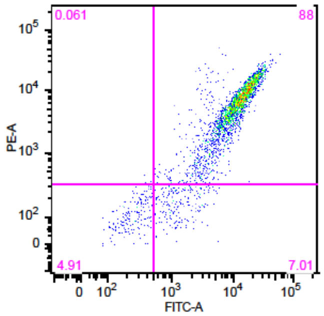

FSC-A, SSC-A subset
Specimen_001_86p.fcs
Event Count: 3281

#90 Untreated

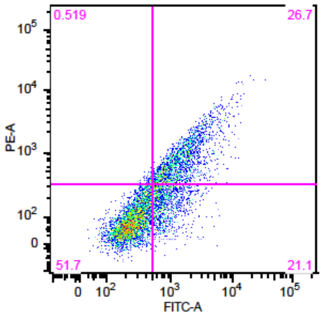

FSC-A, SSC-A subset
Specimen_001_90.fcs
Event Count: 6555

#90 PMA

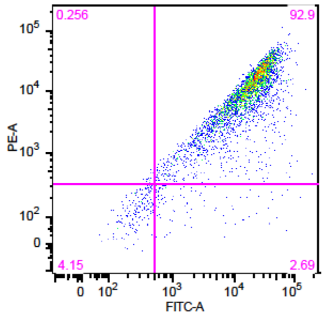

FSC-A, SSC-A subset
Specimen_001_90p.fcs
Event Count: 3901

#91 Untreated

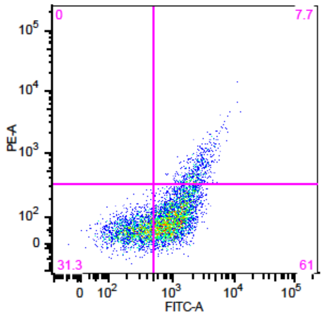

FSC-A, SSC-A subset
Specimen_001_91.fcs
Event Count: 5791

#91 PMA

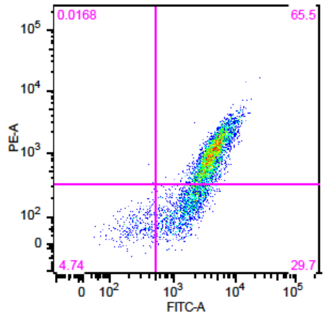

FSC-A, SSC-A subset
Specimen_001_91p.fcs
Event Count: 5968

Supplementary Figure S2 – FACS analysis of cloned parental lines

Class A (Indicated in Figure 2)

#106 Untreated

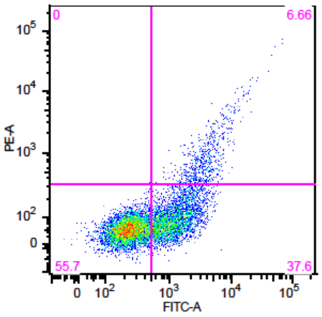

FSC-A, SSC-A subset
Specimen_001_106.fcs
Event Count: 10009

#106 PMA

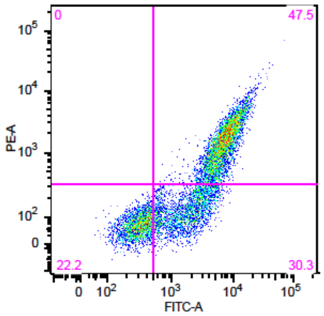

FSC-A, SSC-A subset
Specimen_001_106p.fcs
Event Count: 9979

#115 Untreated

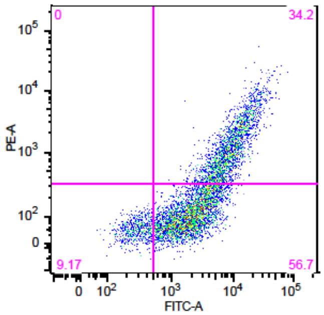

FSC-A, SSC-A subset
Specimen_001_115.fcs
Event Count: 6545

#115 PMA

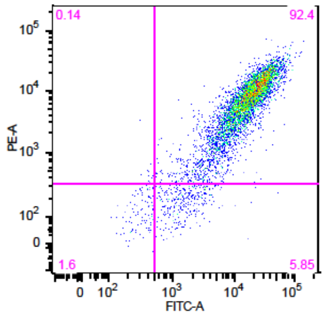

FSC-A, SSC-A subset
Specimen_001_115p.fcs
Event Count: 5696

#118 Untreated

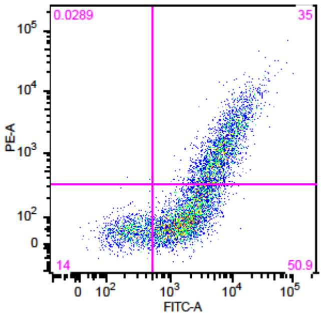

FSC-A, SSC-A subset
Specimen_001_118.fcs
Event Count: 6931

#118 PMA

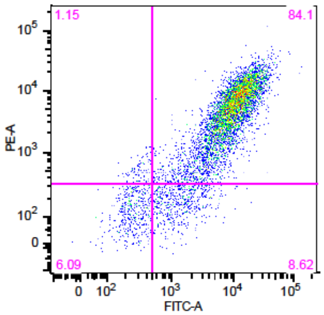

FSC-A, SSC-A subset
Specimen_001_118p.fcs
Event Count: 6671

Supplementary Figure S2 – FACS analysis of cloned parental lines

Class A (Indicated in Figure 2)

#120 Untreated

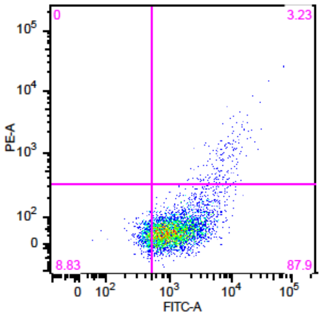

FSC-A, SSC-A subset
Specimen_001_120.fcs
Event Count: 4735

#120 PMA

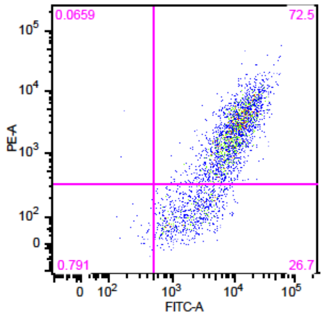

FSC-A, SSC-A subset
Specimen_001_120p.fcs
Event Count: 3034

#121 Untreated

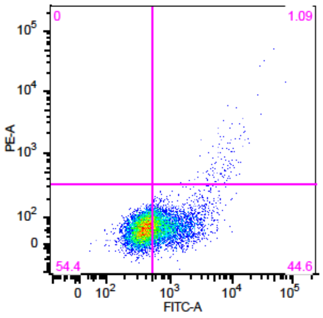

FSC-A, SSC-A subset
Specimen_001_121.fcs
Event Count: 10005

#121 PMA

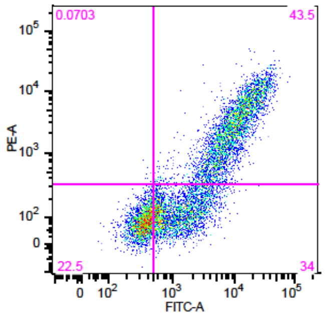

FSC-A, SSC-A subset
Specimen_001_121p.fcs
Event Count: 9956

#122 Untreated

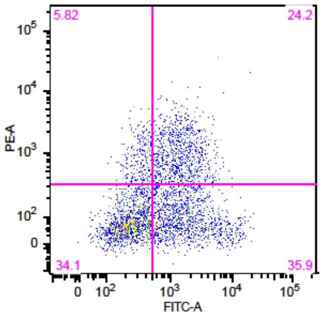

FSC-A, SSC-A subset
Specimen_001_122.fcs
Event Count: 4263

#122 PMA

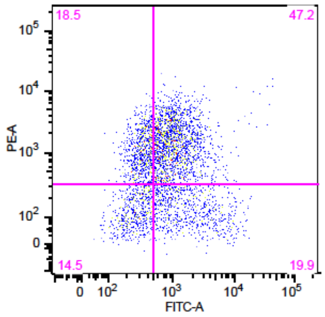

FSC-A, SSC-A subset
Specimen_001_122p.fcs
Event Count: 3742

Supplementary Figure S2 – FACS analysis of cloned parental lines

Class A (Indicated in Figure 2)

#131 Untreated

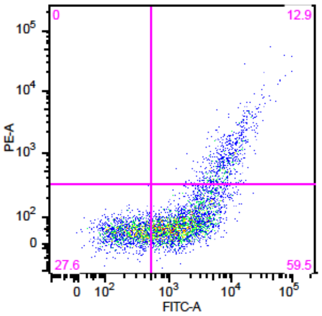

FSC-A, SSC-A subset
Specimen_001_131.fcs
Event Count: 4894

#131 PMA

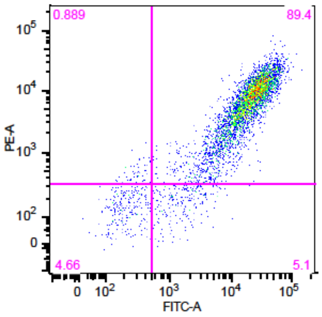

FSC-A, SSC-A subset
Specimen_001_131p.fcs
Event Count: 4725

#134 Untreated

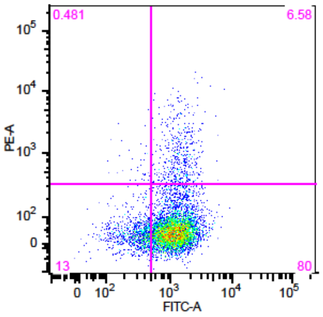

FSC-A, SSC-A subset
Specimen_001_134.fcs
Event Count: 7068

#134 PMA

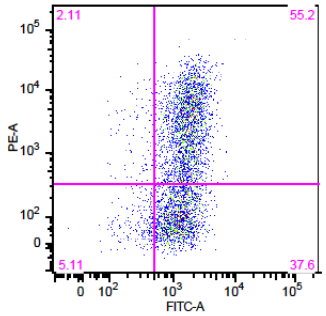

FSC-A, SSC-A subset
Specimen_001_134p.fcs
Event Count: 4406

#141 Untreated

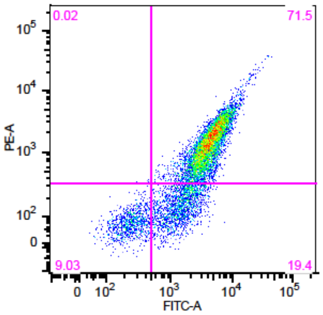

FSC-A, SSC-A subset
Specimen_001_141.fcs
Event Count: 10000

#141 PMA

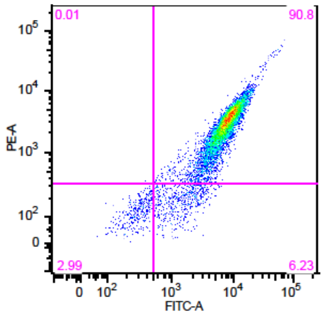

FSC-A, SSC-A subset
Specimen_001_141p.fcs
Event Count: 9988

Supplementary Figure S2 – FACS analysis of cloned parental lines

Class A (Indicated in Figure 2)

#143 Untreated

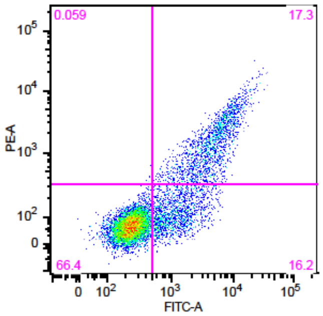

FSC-A, SSC-A subset
Specimen_001_143.fcs
Event Count: 10170

#143 PMA

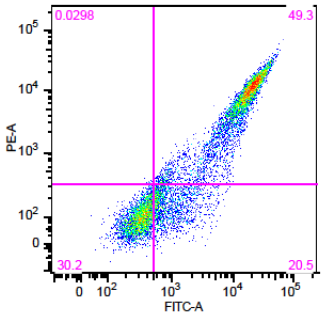

FSC-A, SSC-A subset
Specimen_001_143p.fcs
Event Count: 10054

Supplementary Figure S2 – FACS analysis of cloned parental lines

Class B (Indicated in Figure 2)

#11 Untreated

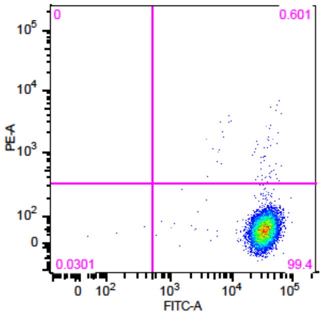

FSC-A, SSC-A subset
Specimen_001_11.fcs
Event Count: 9982

#11 PMA

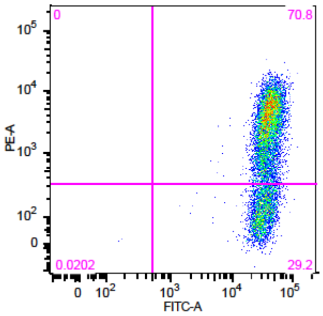

FSC-A, SSC-A subset
Specimen_001_11p.fcs
Event Count: 9919

#80 Untreated

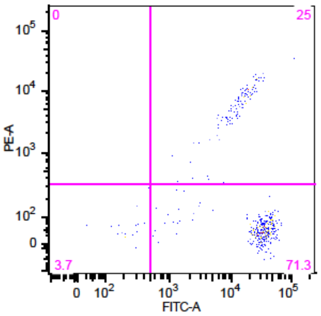

FSC-A, SSC-A subset
Specimen_001_80.fcs
Event Count: 432

#80 PMA

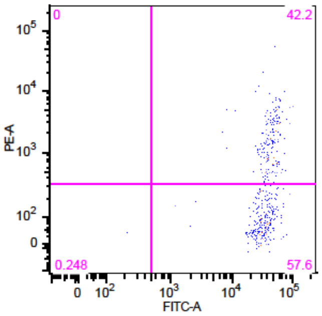

FSC-A, SSC-A subset
Specimen_001_80p.fcs
Event Count: 403

#83 Untreated

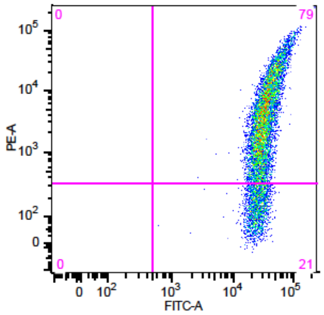

FSC-A, SSC-A subset
Specimen_001_83.fcs
Event Count: 9978

#83 PMA

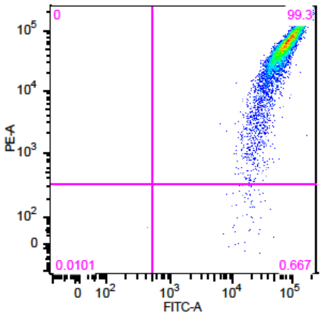

FSC-A, SSC-A subset
Specimen_001_83p.fcs
Event Count: 9901

Supplementary Figure S2 – FACS analysis of cloned parental lines

Class B (Indicated in Figure 2)

#112 Untreated

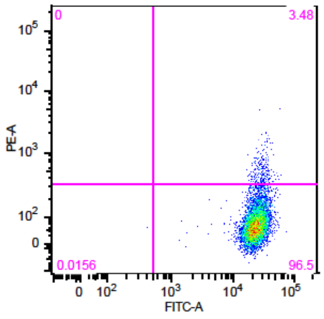

FSC-A, SSC-A subset
Specimen_001_112.fcs
Event Count: 6414

#112 PMA

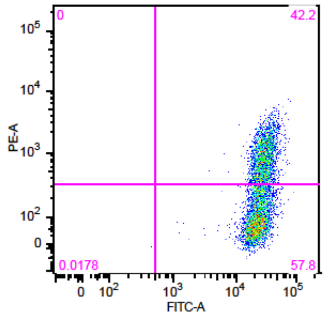

FSC-A, SSC-A subset
Specimen_001_112p.fcs
Event Count: 5625

Supplementary Figure S2 – FACS analysis of cloned parental lines

Class C (Indicated in Figure 2)

#58 Untreated

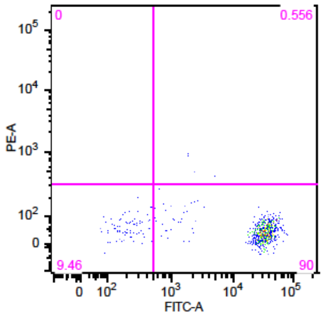

FSC-A, SSC-A subset
Specimen_001_58.fcs
Event Count: 719

#58 PMA

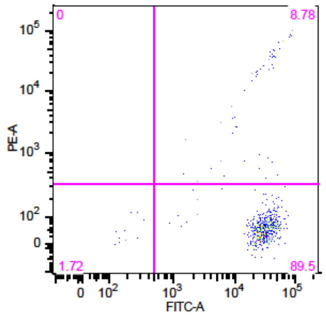

FSC-A, SSC-A subset
Specimen_001_58p.fcs
Event Count: 581

#89 Untreated

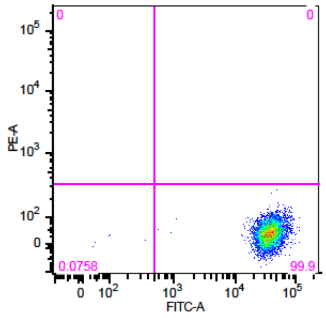

FSC-A, SSC-A subset
Specimen_001_89.fcs
Event Count: 6594

#89 PMA

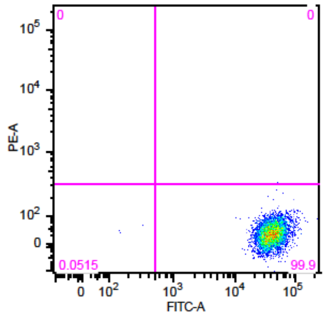

FSC-A, SSC-A subset
Specimen_001_89_p.fcs
Event Count: 5828

#94 Untreated

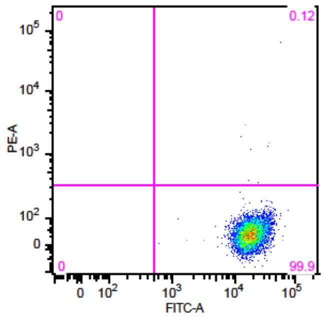

FSC-A, SSC-A subset
Specimen_001_94.fcs
Event Count: 6684

#94 PMA

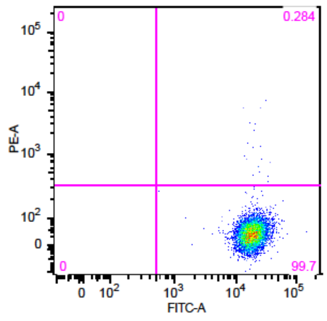

FSC-A, SSC-A subset
Specimen_001_94p.fcs
Event Count: 6693

Supplementary Figure S2 – FACS analysis of cloned parental lines

Class D (Indicated in Figure 2)

#5 Untreated

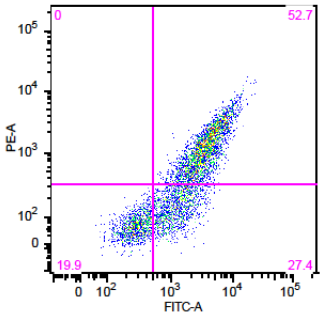

FSC-A, SSC-A subset
Specimen_001_5.fcs
Event Count: 5057

#5 PMA

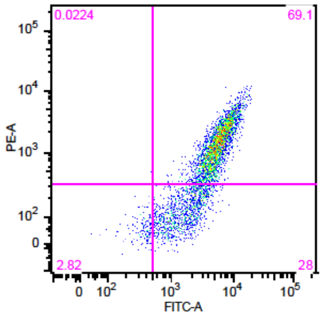

FSC-A, SSC-A subset
Specimen_001_5p.fcs
Event Count: 4462

#25 Untreated

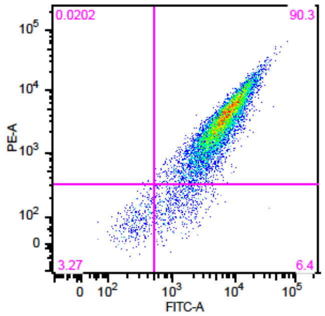

FSC-A, SSC-A subset
Specimen_001_25.fcs
Event Count: 9921

#25 PMA

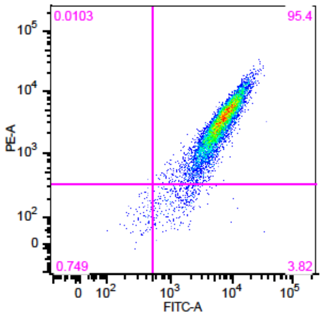

FSC-A, SSC-A subset
Specimen_001_25p.fcs
Event Count: 9747

#35 Untreated

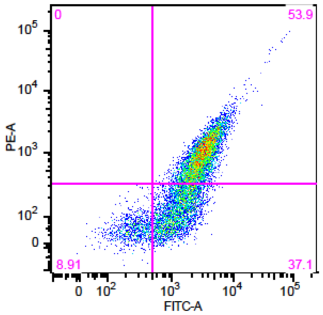

FSC-A, SSC-A subset
Specimen_001_35.fcs
Event Count: 9939

#35 PMA

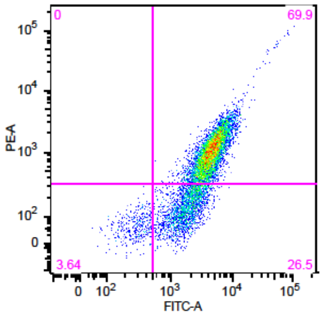

FSC-A, SSC-A subset
Specimen_001_35p.fcs
Event Count: 9804

Supplementary Figure S2 – FACS analysis of cloned parental lines

Class D (Indicated in Figure 2)

#46 Untreated

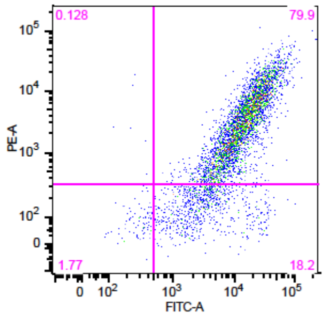

FSC-A, SSC-A subset
Specimen_001_46.fcs
Event Count: 4689

#46 PMA

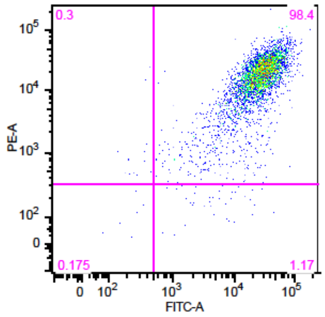

FSC-A, SSC-A subset
Specimen_001_46p.fcs
Event Count: 4003

#53 Untreated

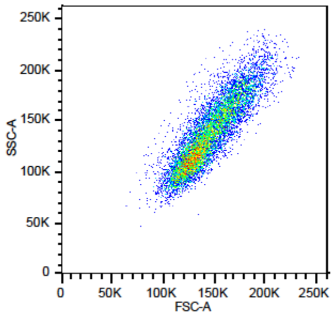

FSC-A, SSC-A subset
Specimen_001_53.fcs
Event Count: 9998

#53 PMA

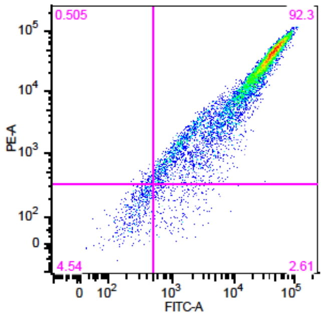

FSC-A, SSC-A subset
Specimen_001_53p.fcs
Event Count: 9909

#60 Untreated

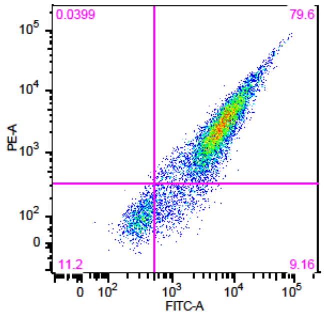

FSC-A, SSC-A subset
Specimen_001_60.fcs
Event Count: 10018

#60 PMA

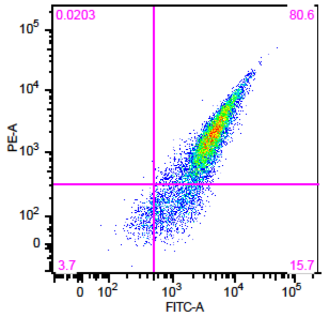

FSC-A, SSC-A subset
Specimen_001_60p.fcs
Event Count: 9836

Supplementary Figure S2 – FACS analysis of cloned parental lines

Class D (Indicated in Figure 2)

#110 Untreated

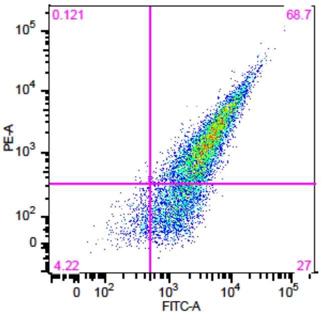

FSC-A, SSC-A subset
Specimen_001_110.fcs
Event Count: 9931

#110 PMA

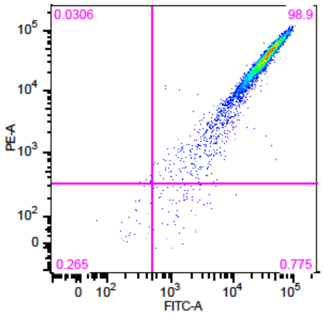

FSC-A, SSC-A subset
Specimen_001_110p.fcs
Event Count: 9807

#144 Untreated

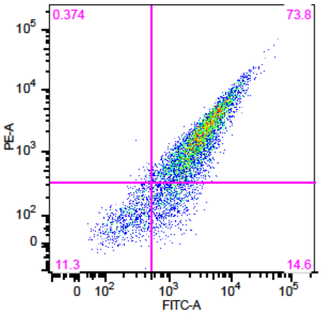

FSC-A, SSC-A subset
Specimen_001_144.fcs
Event Count: 6425

#144 PMA

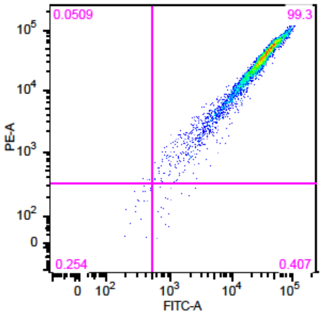

FSC-A, SSC-A subset
Specimen_001_144p.fcs
Event Count: 5895

Supplementary Figure S3 – Comparison of provirus responses to signaling agonists and chromatin modifying compounds.

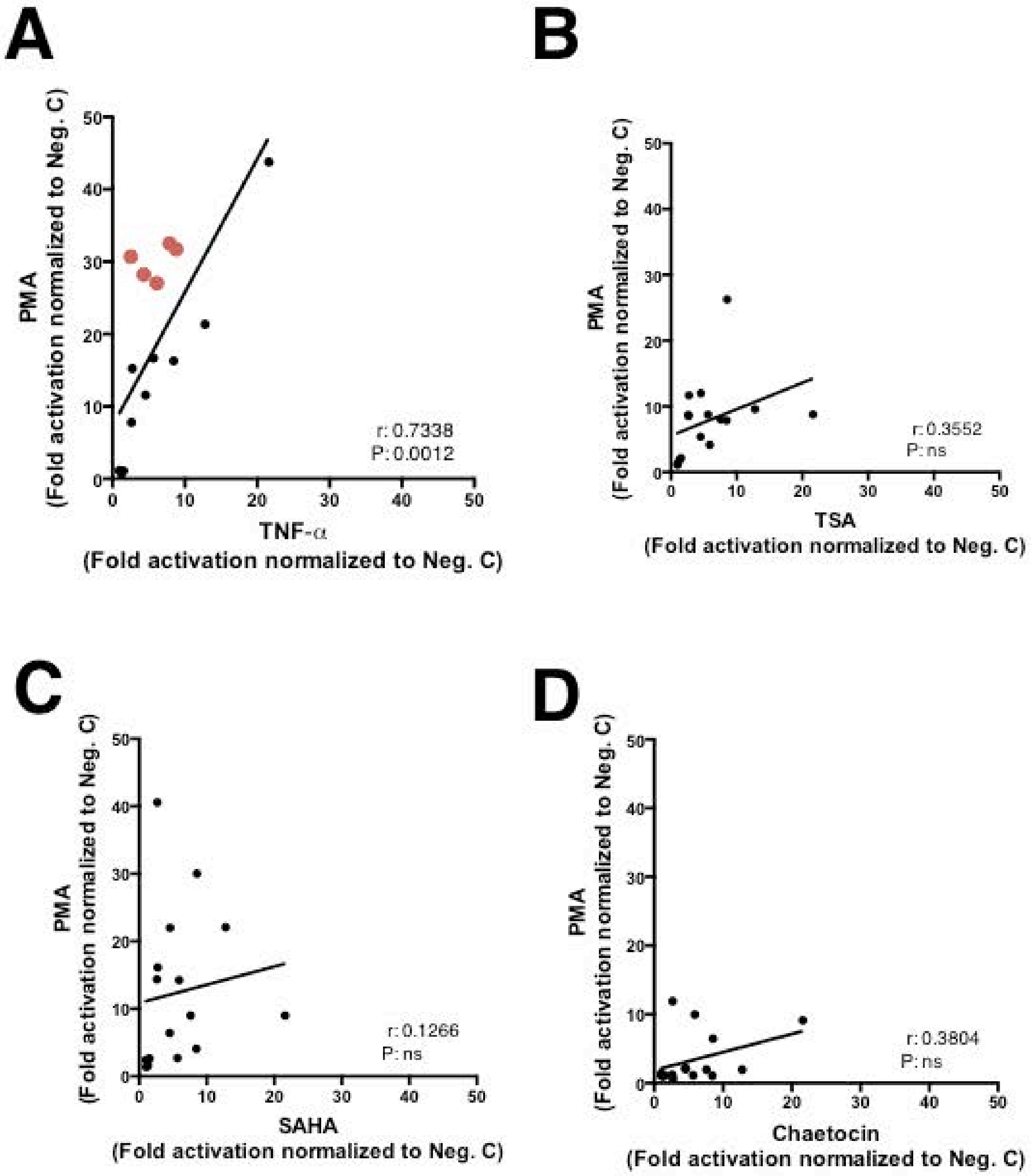

Supplementary Figure S4 – Identification of mdHIV integration sites by nested PCR.

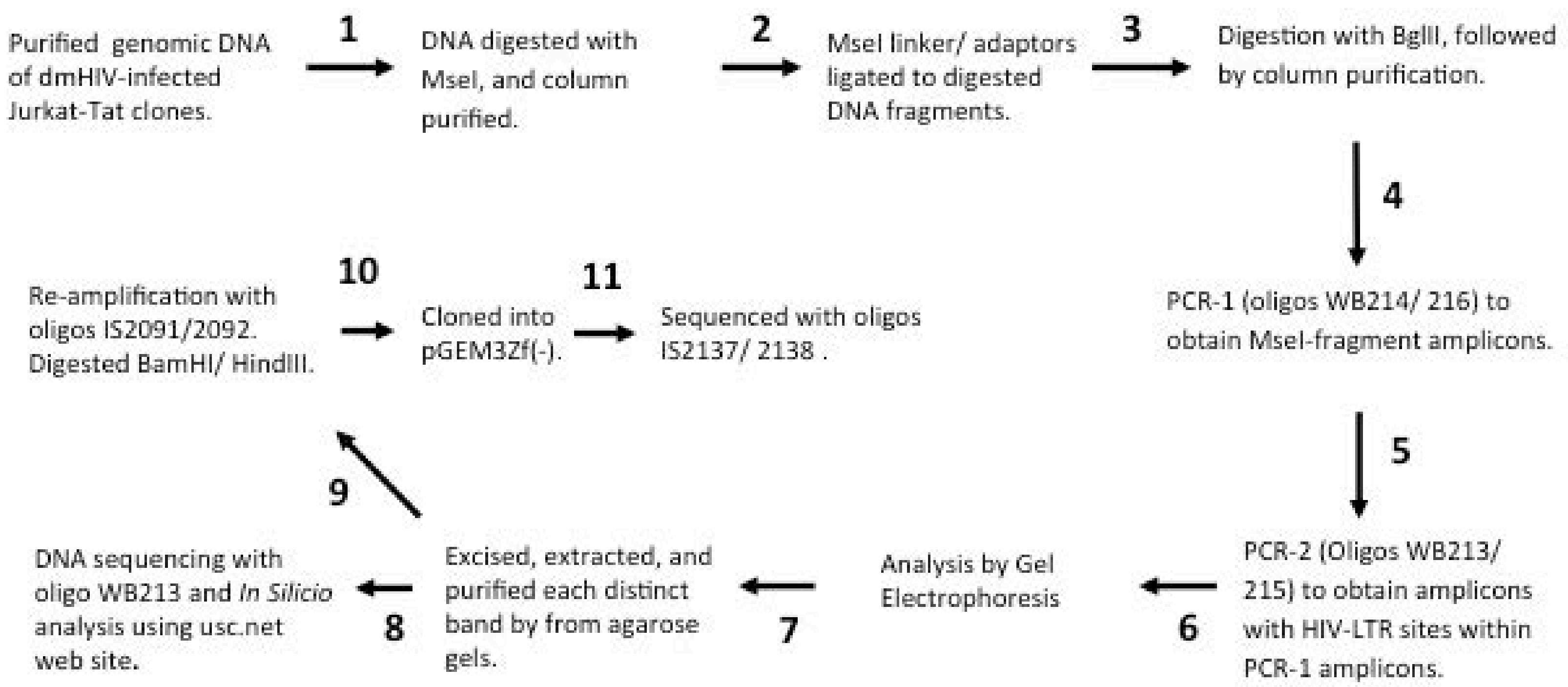

Supplementary Figure S5 – FACS analysis of line 25 and 60 subclones following 6 weeks of culture.

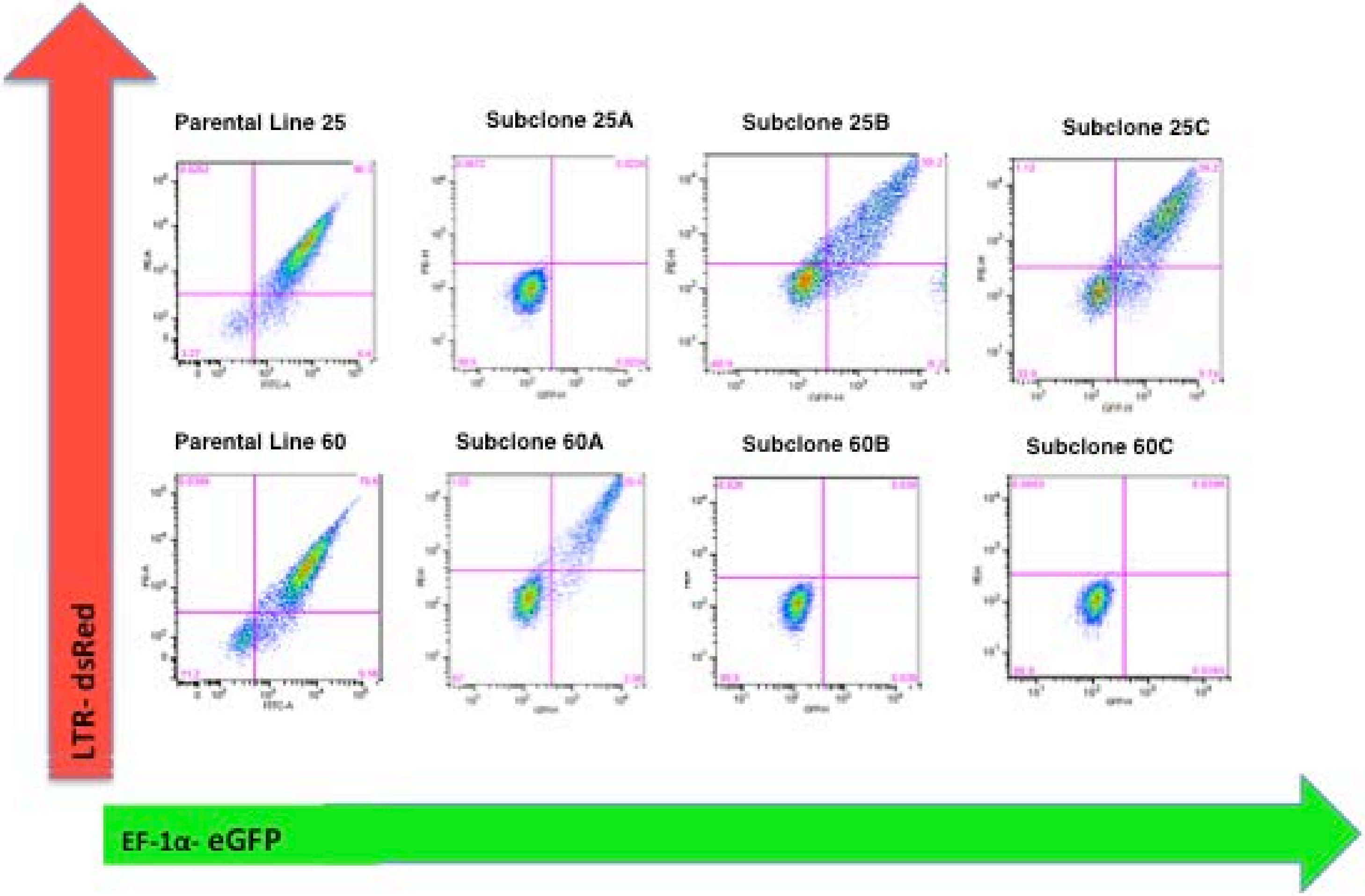

Supplementary Figure S6 – FACS analysis of line 77 subclones following 6 weeks of culture.

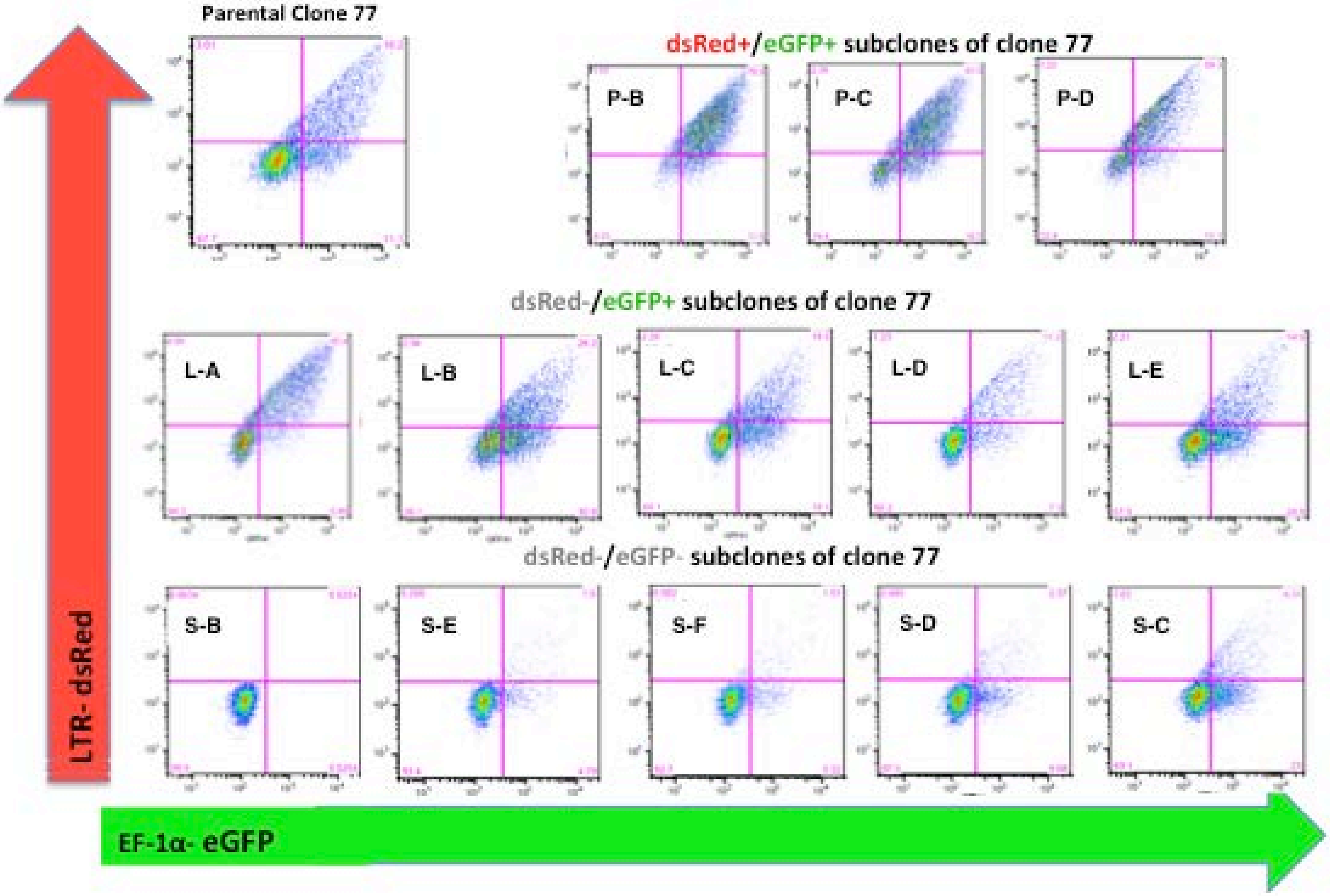

Supplementary Figure S7 – Schematic representation of the PGK Red Green HIV (RGH) reporter virus .

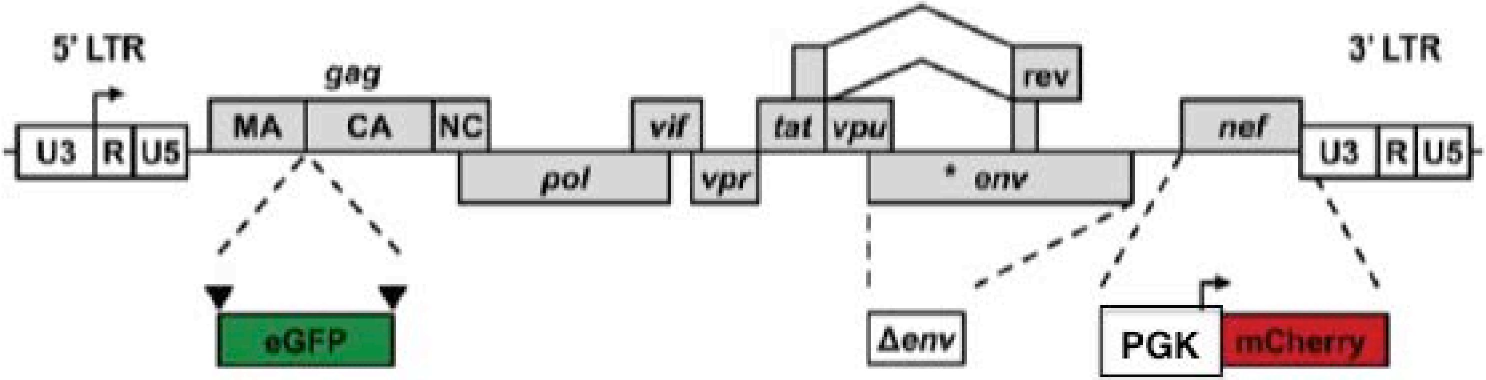

Legends to Supplementary Figures

Supplementary Figure S1. Clonal analysis of mini-dual HIV (mdHIV) provirus integrants. Jurkat Tat cells were infected with mdHIV (**1**) and single infected cells (productive [+/+], or latent[+/-]) were isolated by live soring 24 hours later. Cloned infected cells were expanded in culture for 2 months (**2**), whereupon samples of the cloned populations were analyzed by FACS, either untreated or stimulated with PMA for 24 hours (**3**, Supplementary Figure S2). Representative cloned lines with unique basal and induced GFP and dsRed expression profiles were selected for further analysis (**5**). Integration sites for representative cloned lines was determined (**4**, Figure S4).

Subclones of the parental lines which express both GFP and dsRed (Productive infection +/+), express only dsRed (Latent infection, -/+) or where both reporters had been silenced (Silenced infection, -/-) were isolated by live sorting (**6**) and expanded in culture for a total of 8 weeks (**7**). Expression of GFP and dsRed was analyzed in untreated cultures or following treatment with PMA (**8**), and the expression profiles compared to that produced by the parental cloned cell lines (**9**).

Supplementary Figure S2. FACS analysis of cloned parental Jurkat TAT cells bearing integrations of the mdHIV reporter virus, after 8 weeks expansion in culture. Cells were untreated (left panels), or treated with PMA for 24 hours prior to analysis of GFP (horizontal) or dsRed (vertical) expression by FACS. The

identity of the cloned lines are indicated as "Specimen_001_X.fcs", where X = line designation number. Event count represents the total number of cells analyzed.

Supplementary Figure S3. Panel A. Comparison of fold induction of HIV LTR dsRed expression in response to PMA (vertical) and TNF-α (horizontal) for the cloned provirus cell lines (results from Figure 3A). Results for lines 131, 49, 90, 61 and 53 are indicated in red. Comparison of fold induction of LTR dsRed expression in response to PMA with TSA (**Panel B**), SAHA (**Panel C**), and chaetocin (**Panel D**) for the provirus cell lines (results from Figure 3B).

Supplementary Figure S4. Identification of provirus integration sites in the cloned lines. Genomic DNA was digested with MseI (**1**) and ligated to adaptor oligos (**2**). Samples were subsequently digested with BglII (**3**) and then amplified with MseI adaptor-specific oligos WB214 and WB216 (**4**). The products were reamplified with linker and HIV LTR-specific primers WB213 and WB215 (**5**) and analyzed by electrophoresis (**6**). Individual amplification products were purified from the gel (**7**) and sequenced with oligo WB213 (**8**). Amplification products which did not produce clean sequencing results were re-amplified with oligos IS2091 and IS2092 (**9**), digested with BamHI and HindIII, cloned into pGEM3zf(-) (**10**) and sequenced with primers IS2137 and IS2138 (**11**).

Supplementary Figure S5. Analysis of dsRed and GFP expression patterns in subclones of parental lines 25 and 60. Subclones of parental lines 25 and 60 that express both dsRed and eGFP were expanded in culture for 6 weeks and analyzed by FACS for expression of LTR-dsRed (vertical) or EIF1-α-eGFP (horizontal) expression.

Supplementary Figure S6. Analysis of dsRed and GFP expression patterns in subclones of parental line 77. Subclones of parental line 77 that express both dsRed and eGFP (Productive infection, P-B, P-C and P-D), express eGFP but not dsRed (Latent infection, L-A, L-B, L-C, L-D and L-E), or where both reporters had been silenced (S-B, S-C, S-E, S-D, and S-F) were expanded in culture for 6 weeks and analyzed by FACS for expression of LTR-dsRed (vertical) or EIF1-α-eGFP (horizontal) expression.

Supplementary Figure S7. Expression from the 5' LTR is detected by production of eGFP, produced as a polyprotein precursor with the viral *gag* gene products. Infected cells can be detected independently from LTR activity by expression of mCherry from the PGK promoter, inserted within the *Nef* viral gene.
